# How Trustworthy is Light Transmittance Platelet Aggregometry With Low Platelet Count Samples? Insights From Test Replicates and Retrospective Analysis of Several Decades of Diagnostic Samples

**DOI:** 10.1111/ijlh.14518

**Published:** 2025-06-23

**Authors:** Catherine P. M. Hayward, Rabab Al Dawood, Lindsay Wice, Karen A. Moffat

**Affiliations:** ^1^ Department of Pathology and Molecular Medicine McMaster University Hamilton Canada; ^2^ Department of Medicine McMaster University Hamilton Canada; ^3^ Special Coagulation Laboratory Hamilton Regional Laboratory Medicine Program Hamilton Canada; ^4^ Department of Pathology and Laboratory Medicine King Fahad Specialist Hospital Dammam Saudi Arabia

**Keywords:** platelet aggregation, platelet function tests, thrombocytopenia

## Abstract

**Introduction:**

Light transmission platelet aggregometry (LTA) is useful to diagnose platelet function disorders (PFD). We evaluated the precision and reproducibility of LTA with low platelet count platelet‐rich plasma (LPRP).

**Methods:**

LPRP maximal aggregation (MA) precision for informative agonists and LTA reproducibility were assessed using multiple replicates for adjusted control LPRP (CLPRP) and retrospective analysis of several decades of consecutive patient LPRP (PLPRP) and CLPRP tests with replicates between and/or within tests.

**Results:**

Intra‐subject CLPRP had acceptable MA CVs and tracings unless platelets were ≤ 25 × 10^9^/L. Among tests with replicates, PLPRP with ≤ 25 × 10^9^ platelets/L were uncommon (6/247 samples) and 5/6 showed pathognomonic Bernard–Soulier syndrome (BSS) findings. Among evaluated patients (*n* = 195; diverse diagnoses), 44.6% had abnormal LTA findings on ≥ 1 tests after excluding single, within‐test MA outliers in 21/194 PLPRP and 2/27 CLPRP tests. Median, intra‐subject, within‐test coefficients of variation (CVs) for PLPRP and CLPRP MA responses to informative agonists were acceptable for most agonists, with higher CV for 0.5 mg/mL ristocetin, weak agonists, and impaired responses, and only small differences between MA estimates across agonists. Between‐test agreement was good for MA responses, with 80.5% of patients and 98.1% of controls having consistent overall LTA findings, with significantly more patients than controls having consistently abnormal LTA findings (46.3% vs. 0%; *p* < 0.001).

**Conclusions:**

Diagnostic LTA with LPRP has acceptable precision and reproducibility when evaluated with informative agonists. Nonetheless, caution is warranted when testing samples with ≤ 25 × 10^9^ platelets/L, which often show BSS findings.

## 1 Introduction

Aggregation tests are important to “rule‐in,” and “rule–out,” platelet function disorders (PFD), including PFD associated with thrombocytopenia [1, 2]. While some thrombocytopenic disorders (e.g., *MYH9*‐related disorders, MYH9RD) can have normal aggregation findings, others have pathognomonic maximal aggregation (MA) abnormalities, including congenital Bernard–Soulier syndrome (BSS), *ITGA2/ITGB3*‐related thrombocytopenia (*ITGA2/ITGB3‐*RT), type 2B von Willebrand disease (VWD) and platelet‐type VWD (PT‐VWD) [2, 3, 4]. Additionally, abnormal MA with ≥ 2 agonists is typical of some other thrombocytopenic PFD, including familial platelet disorder with predisposition to myeloid malignancies (FPDMM), acquired PFD complicating hematological malignancies, and immune thrombocytopenia (ITP) with acquired, autoantibody‐induced Glanzmann thrombasthenia (GT) [1, 2, 3].

Low sample platelet counts are known to influence platelet aggregation responses in light transmittance aggregometry (LTA), whole blood aggregometry (WBA), and flow cytometry tests and some agonists are non‐informative for low platelet count samples [1, 2, 3, 5, 6, 7, 8, 9, 10, 11, 12, 13, 14, 15, 16, 17]. For example, LTA MA responses for low platelet count platelet‐rich plasma (LPRP) containing ≤ 140 × 10^9^ platelets/L are informative for ristocetin and high concentrations of collagen and ADP, but are non‐informative for weak agonists and arachidonic acid (AA) [1, 2]. Additionally, LPRP MA responses to ristocetin and high concentrations of Horm collagen (a strong agonist) are evaluable, even for samples with ≤ 80 × 10^9^ platelets/L, using LPRP MA reference intervals (RI) for the sample platelet count to guide interpretations [1, 2]. Nonetheless, there is limited information on the trustworthiness of diagnostic LTA with LPRP. This is an important consideration as ~12%–17% of all patient diagnostic LTA samples are LPRP [1, 2]. Some studies of diluted control samples (CLPRP) suggest that LTA precision is poor when sample platelet counts are 10 or 50 × 10^9^/L, based on MA responses to 6.5 μM ADP and 32 μM PAR1 activating peptide [13].

The Hamilton Regional Laboratory Medicine Program (HRLMP) Special Coagulation Laboratory standard operating procedure is to run the patient LPRP (PLPRP) in parallel with a platelet count‐matched, normal control PRP sample (CLPRP), prepared by dilution with autologous platelet poor plasma (PPP) [1, 2]. It also requires that tests be done with agonists, and agonist concentrations, that are validated to be informative for LTA with LPRP, with omission of AA and thromboxane analog U46619 for LPRP with ≤ 140 × 10^9^ platelets/L, and omission of weak agonists (such as ADP and low concentrations of Horm collagen) for LPRP with ≤ 80 × 10^9^ platelets/L [1, 2]. Furthermore, the procedures require LPRP MA to be classified as normal or abnormal, based on LPRP MA RI that were derived from regression analysis of CLPRP tested over a broad range of low platelet counts [1]. We recently validated this diagnostic approach with a second patient cohort, with updates on which agonists to test and how to verify, interpret, and report abnormal findings [2]. While CLPRP rarely have abnormal MA findings, ~25% of consecutive PLPRP have MA abnormalities, which rises to ~60% for PLPRP with ≤ 80 × 10^9^ platelets/L [2]. Furthermore, ~36% of PLPRP abnormalities can be classified as true positives based on other confirmatory tests (i.e., a confirmed pathogenic PFD or type 2B VWD genetic mutation; a platelet glycoprotein deficiency consistent with inherited BSS or suspected *ITGA2/ITGB3*‐RT by flow cytometry) [2].

An important consideration for the precision of LTA is the within‐subject MA CVs, which for normal platelet count samples, are <10% for most agonists, higher for weak agonists (~15% for 2.5 μM ADP, < 10 ~ 27% for 6–10 μM epinephrine), and highest for 0.5 mg/mL ristocetin (59%) due to the limited response (MA: ~0%–7%) [18, 19]. Furthermore, >90% of patients with inherited mucocutaneous bleeding disorders, assessed by LTA with normal platelet count samples, have normal or abnormal findings confirmed on repeat aggregation tests [19].

The primary goal of this study was to assess the reliability and precision of LPRP LTA using:

1. HRLMP quality assurance (QA) data on multiple intra‐subject, within‐test CLPRP replicates with informative agonists, to determine how sample platelet counts affect MA precision and the acceptability of LTA findings and tracings for interpretation.
2. 28 years of HRLMP LPRP LTA records for unbiased information on:
  - Technical problems (including rejections) for PLPRP with extremely low platelet counts (i.e., ≤ 25 × 10^9^/L).
  - The prevalence of outlier MA among CLPRP and PLPRP tests with informative agonists.
  - The variability of intra‐subject MA estimates (within and between tests, after excluding within‐test outliers, after adjudication) for consecutive PLPRP and CLPRP tests with replicates.
  - The intra‐subject agreement in whether LPRP MA responses to agonists and overall LTA findings were consistently normal or abnormal between tests for patients and controls.

## 2 Methods

The study was conducted with approval of the Hamilton Integrated Research Ethics Board (HIREB project 18602) and in accordance with the revised Helsinki Declaration on human research.

HRLMP aggregation tests were done on PACKS‐4 (until April 2008) and then AggRAM analyzers, both from Helena Laboratories (Beaumont, Texas), with validated equivalence of the measured aggregation [1, 20]. LTA was tested using HRLMP procedures and agonists (sources of agonists and other details as previously described) [1, 2, 21]. Data were collected for agonists considered informative for the sample LPRP platelet count [2], as summarized below:

1. 240–249 × 10^9^/L: 2.5 and 5 μM ADP, 1.25 and 5.0 μg/mL Horm collagen (collagen), 1.6 mM AA, 1 μM thromboxane analog U46619 (U46619), 0.5 and 1.25 mg/mL ristocetin, and 6 μM epinephrine.
2. 141–239 × 10^9^/L: all above agonists except epinephrine.
3. 81–140 × 10^9^/L: 5 μM ADP, 5.0 μg/mL collagen and 0.5 and 1.25 mg/mL ristocetin.
4. ≤ 80 × 10^9^/L: 5.0 μg/mL collagen and 0.5 and 1.25 mg/mL ristocetin.

LTA RI for the PACKS‐4 instrument [1] were locally validated for transference to the AggRAM instrument by:

1. parallel testing of 20 control PRP adjusted to 250 × 10^9^ platelets/L, with all agonists (including: 3 controls tested in triplicate/agonist on the AggRAM and 16 controls tested in parallel with all agonists on AggRAM modules A and B), and
2. analysis of consecutive CLPRP, tested on the AggRAM with informative agonists between May 2008 and January 2022 that verified 95.5–100% of CLPRP MA responses were within published RI (65–179 MA estimates evaluated/agonist).

QA data were used to assess the effects of sample platelet counts on the precision of CLPRP MA responses (based on 8 within‐test/within‐subject replicates/sample) to 5.0 μg/mL collagen and 0.5 and 1.25 mg/mL ristocetin (i.e., the agonists informative for all LPRP with ≤ 80 to 249 × 10^9^ platelets/L), using samples adjusted with autologous PPP to final counts of: 250, 75, 50, 25, 20, 15, and 10 × 10^9^ platelets/L. Within‐test MA CVs and ranges of MA estimates were determined.

HRLMP paper copies of LTA records from January 1997 to March 10, 2025 (which contained information on age and sex and complete blood counts [CBC]) were retrospectively examined for PLPRP and CLPRP MA replicates with informative agonists and any technical problems (including rejections) with LPRP tests, particularly for very low platelet count LPRP (e.g., ≤ 25 × 10^9^ platelets/L). We evaluated all consecutive historical LPRP records, for patients and controls that met the inclusion criteria of having within‐test MA replicates with ≥ 1 agonist and/or data for multiple LPRP tests. Data were excluded for rejected controls and for samples showing pre‐analytical interference from lipemia or from leukocyte contamination (defined as having impaired MA for LPRP with > 0.4 × 10^9^ leukocytes/L but normal findings for LPRP with < 0.4 × 10^9^ leukocytes/L). Age in years at time of first LTA test was younger than reported in [2] if there were earlier records. Within‐test outlier MA results (identified by CPMH, verified by KAM for consensus) were excluded before determining within‐subject MA CVs, and ranges for MA values within replicates (within and between tests) for informative agonists. Overall test findings (for patients and controls) were classified as abnormal if there was reduced MA with multiple agonists and/or abnormal findings with ristocetin from BSS or VWD, and as normal/non‐diagnostic if all MA were within RI or if only one agonist response was abnormal (often: reduced MA with U46619) [2].

Electronic medical reviews were conducted (CPMH) to gather information on diagnoses and other test findings.

### 2.1 Statistical Analysis

CVs and range of MA estimates for replicates were evaluated to assess intra‐subject MA precision within tests and between tests for CLPRP and PLPRP. Samples with 141–249 × 10^9^ platelets/L were analyzed as one group as few LPRP contained 240–249 × 10^9^ platelets/L. Proportions were evaluated by Chi‐Square tests. Two‐tailed Mann–Whitney tests were used to compare two groups of data. Pearson correlation coefficients were used to assess continuous variables for associations. Statistical significance was defined as *p* values below 0.05.

## 3 Results

### 3.1 Precision and Acceptability of Very Low Platelet Count Samples Based on Quality Assurance (QA) Data

QA data for intra‐subject CLPRP LTA responses, assessed for 4 representative controls, *n* = 3 with samples tested at 10, 15, 20, 25, 50, 75, and 250, and *n* = 1 tested at 25 × 10^9^ platelets/L (8 replicates/agonist/sample), indicated that MA responses to 1.25 mg/mL ristocetin and 5 μg/mL collagen were lower, with MA CVs exceeding 20% for some samples with ≤ 25 × 10^9^ platelets/L (Table 1; Table S1 shows all estimates). MA CVs were considerably higher for 0.5 mg/mL ristocetin, across all samples (Table 1). Additionally, MA estimates for some samples with ≤ 50 × 10^9^ platelets/L, tested with collagen and ristocetin, were outside published RI [1] (Table 1). Aggregation tracings for CLPRP became difficult to evaluate when CLPRP counts were ≤ 25 × 10^9^ platelets/L (Figure S1).

**TABLE 1:** Effect of platelet‐rich plasma platelet counts on the within‐test precision of maximal aggregation responses to informative agonists evaluated using quality assurance data for control samples. Data are summarized for the endpoints of percentage maximal aggregation, and differences between percentage maximal aggregation estimates for replicates (8 each/test at each sample platelet count) as medians, and median CVs. Four controls had 8 replicates/agonist at 25 × 10^9^ platelets/L and three had 8 replicates/agonist for all other sample platelet counts.

	Median %MA (range) Median MA CV [range] with			Median difference in %MA (range), Median CV for differences in %MA with		
Sample platelet count	5 μg/mL collagen	1.25 mg/mL ristocetin	0.5 mg/mL ristocetin	5 μg/mL collagen	1.25 mg/mL ristocetin	0.5 mg/mL ristocetin
250 × 10^9^/L	94.5 (87–97) 1.4 [0.9–2.2]%	85 (76–94) 4.6 [2.3–6.2]%	2.5 (1–7) 51 [45–52]%	4 (3–6) 28.8%	11 (6–18) 42.2%	4 (3–5) 20.4%
75 × 10^9^/L	82.5 (58–91) 3.2 [2.9–3.8]%	82 (73–89) 3.4 [2.7–4.6]%	3 (1–9) 49 [48–64]%	8 (6–10) 20.4%	9 (7–11) 18.1%	6 (3–7) 31.9%
50 × 10^9^/L	73 (36–81)^a^ 2.6 [2.4–5.9]%	69 (47–87)^a^ 3.9 [3.4–15]%	6 (1–17) 39 [22–94]%	7 (5–8) 18.7%	10 (7–40) 78.4%	6 (5–16) 55.2%
25 × 10^9^/L	39.5 (5–62)^a^ 7.2 [5.5–68]%	50.5 (36–71)^a^ 8.3 [6.0–11]%	7 (1–32)^a^ 41 [31–56]%	12.5 (6–45) 80.4%	13 (11–17) 16.1%	9 (7–20) 45.9%
20 × 10^9^/L	30.5 (13–54)^a^ 26 [12–70]%	35.5 (25–91)^a^ 15 [14–38]%	10 (2–25)^a^ 54 [39–62]%	23 (14–44) 46.6%	16 (16–57) 65.1%	20 (13–23) 22.4%
15 × 10^9^/L	22.5 (11–51) 31 [23–42]%	23 (7–47)^a^ 24 [21–44]%	18 (3–43)^a^ 47.5 [27–57]%	19 (17–35) 34.0%	22 (7–32) 50.5%	29 (13–34) 35.4%
10 × 10^9^/L	22 (12–81)^a^ 34 [23–64]%	14 (6–43)^a^ 41 [26–46]%	22 (8–53)^a^ 36 [28–56]%	34 (12–69) 61.2%	18 (10–32) 45.5%	34 (23–35) 17.7%

Abbreviations: CV, coefficient of variation; MA, maximal aggregation.

^a^ indicates some or all MA values for samples tested at that platelet count were abnormal, based on published reference ranges [1].

### 3.2 Details for Consecutive Patients and Controls That Met Inclusion Criteria

Among the 28 years of LTA records, 195 patients (127 females, 68 males; 1–4 samples/person; median age 40 years, range 0.5–91 years, inter‐quartile range [IQR] 25–58 years), with 247 PLPRP tests, met the inclusion criteria of having within‐test MA replicates for ≥ 1 agonist(s) and/or multiple tests performed. One patient (P50644 from [2]) was excluded for leukocyte contamination affecting 2 of 3 samples tested.

Among patients, diagnoses were diverse (Table 2, Table S2). Seventy‐one were previously unreported patients, whereas 37 were reported in [1] and 87 in [2]. Among those reported in [2], proportionately more with abnormal than non‐diagnostic LTA findings met inclusion criteria (41/45 vs. 46/147, *p* < 0.01). Historical data were available for 57 unique controls (48 females, 8 males, 2 sex unknown; *n* = 27 with “within‐test” MA replicates for ≥ 1 agonist; *n* = 53 with multiple CLPRP tests, including *n* = 7 with ≥ 10–41 CLPRP tests) (Table S3).

**TABLE 2:** Diagnoses for the 195 evaluated patients with maximal aggregation replicates for low platelet count samples. Information summarized by diagnoses includes the percentages of cases with abnormal LTA findings and tested at different sample platelet count ranges.

			% tested with informative agonists for LPRP with platelet counts of			
Diagnosis, number of cases	% of all cases	% abnormal with multiple agonists &/or ristocetin on ≥ 1 tests	≤ 80 × 10^9^/L	81–140 × 10^9^/L	141–239 × 10^9^/L	240–249 × 10^9^/L
Immune thrombocytopenia, *n* = 45 (*n* = 4 with acquired BSS and/or GT)	23.1%	38%	24%	38%	36%	2%
Thrombocytopenia of unknown cause, *n* = 41 (*n* = 21 familial, *n* = 1 with HHT)	21.0%	39%^d^	12%	32%	54%	2%
Non‐diagnostic bleeding disorder investigations, *n* = 14 (*n* = 2 with BUC)	7.2%	0%			86%	14%
Thrombocytopenia from liver disease, *n* = 14 (*n* = 1 with type 2 M VWD)	7.2%	29%	7%	29%	65%	
Bernard–Soulier syndrome, *n* = 10 (*n* = 1 with liver disease)	5.6%	100%	90%	10%		
Hematological disorder without evidence of a PFD, *n* = 7 (*n* = 6 with MDS, *n* = 1 with CML)	3.6%	0%	14%	43%	43%	
Type 2B VWD, *n* = 6	3.1%	100%	83%	17%		
Acquired PFD complicating MDS or a lymphoproliferative disorder, *n* = 5 (*n* = 1 with a pathogenic *RUNX1* mutation, *n* = 4 with DGD)	2.1%	80%^a^	20%	20%	60%	
PFD, unknown cause, *n* = 10 (*n* = 1 with a *RUNX1* VUS, *n* = 1 with non‐syndromic DGD)	2.6%	100%	12.5%	12.5%	75%	
Suspected platelet‐type VWD, *n* = 2		100%			50%	50%
Familial platelet disorder with predisposition to myeloid malignancy, *n* = 3	1.5%	100%			100%	
Suspected *ITGA2/ITGB3*‐RT, *n* = 2	1%	100%	100%			
Suspected MYH9RD, *n* = 2	1%	0%	100%			
Gray platelet syndrome, *n* = 2	1%	0%	50%	50%		
Phospholipid antibody syndrome, *n* = 2	1%	100%		50%	50%	
Gestational thrombocytopenia, *n* = 2	1%	50%^c^	50%		50%	
NSAID‐induced defects, *n* = 2 (*n* = 1 with radiation‐induced vasculopathy)	1%	100%			100%	
Quebec platelet disorder, *n* = 1	0.5%	0%			100%	
Jacobsen syndrome, *n* = 1	0.5%	0%			100%	
*TUBB1*‐related thrombocytopenia, *n* = 1	0.5%	100%		100%		
Type 2 M von Willebrand disease, *n* = 1 with delayed RIPA, normal platelet count	0.5%	100%			100%	
Other thrombocytopenias,^b^ *n* = 4	1.5%	50%	25%	25%	50%	
Records not available, *n* = 18	9.2%	28%		39%	55%	6%

Abbreviations: BSS, Bernard–Soulier syndrome; BUC, bleeding of unknown cause; CML, chronic myeloid leukemia; DGD, dense granule deficiency; GT, Glanzmann thrombasthenia; HHT, hereditary hemorrhagic telangiectasia; *ITGA2/ITGB3* RT, *ITGA2/ITGB3*‐related thrombocytopenia; ITP, immune thrombocytopenia; MDS, myelodysplastic syndrome; MYH9RD, *MYH9*‐related disorder; PFD, platelet function disorders; RIPA, ristocetin‐induced platelet agglutination/aggregation; VUS, variant of uncertain significance.

^a^ One patient with an acquired platelet function disorder had confirmed dense granule deficiency but non‐diagnostic aggregation findings.

^b^ Other thrombocytopenias were (*n* = 1 for each): drug‐induced, secondary to blood loss, secondary to non‐hematological malignancy, or transient.

^c^ One patient with gestational thrombocytopenia had a suspected false positive with 1.25 mg/mL ristocetin.

^d^ Two patients with thrombocytopenia of unknown cause had abnormal aggregation findings due to a non‐steroidal anti‐inflammatory drug that inhibits cyclooxygenase 1.

Among patients, PLPRP platelet counts were generally higher and showed significant association to CBC platelet counts (Table S2; r^2^ 0.65, *p* < 0.001; respective values, ×10^9^/L as medians [ranges]: 146 [9–247] vs. 89 [4–279]). Only 2.1% had PLPRP with ≤ 25 × 10^9^ platelets/L.

### 3.3 Acceptability of Clinical Samples With Very Low Platelet Counts for LTA

Over 28 years, only 3 PLPRP (< 1% of all PLPRP tested during that period) had been rejected for poor platelet recovery (platelet counts, ×10^9^/L: PLPRP ≤ 11 vs. 25–70 in blood vs. 29–62 for subsequent gravity separated PLPRP; *n* = 1 BSS; *n* = 2 suspected *ITGA2/ITGB3*‐RT, one rejected because of difficult‐to‐interpret LTA tracings, similar to CLPRP with 10 × 10^9^ platelets/L). However, all other PLPRP with ≤ 25 × 10^9^ platelets/L (*n* = 4; range: 9–19 × 10^9^ platelets/L), and the rejected BSS sample, showed pathognomonic BSS findings (Figure S2), like the congenital BSS samples with higher platelet counts (*n* = 8, with 36–102 × 10^9^ platelets/L). In four, the cause was congenital BSS (verified by flow cytometry), and in the other, the cause was acquired BSS complicating ITP, with absent ristocetin‐induced platelet aggregation (RIPA) evident before and after intravenous gammaglobulin treatment (post‐treatment PLPRP with 63 × 10^9^ platelets/L). Accordingly, all PLPRP with ≤ 25 × 10^9^ platelets/L, except the rejected suspected *ITGA2/ITGB3*‐RT PLPRP, were included in analyses, along with PLPRP with higher platelet counts.

### 3.4 Within‐Test Maximal Aggregation Outliers for Patient and Control Samples

In total, 7.4% (2/27) of controls (Table S3) and 10.8% (21/194) of patients (Table S2) had a single MA outlier, for just one agonist, in within‐test replicates. Most outliers were single false positives (20/23) and others were discrepant normal or abnormal results (values highlighted in Tables S2, S3).

Following outlier exclusion, 44.6% (87/195) of patients were considered to have abnormal LTA findings with multiple agonists and/or ristocetin on ≥ 1 tests (Table 2). Most patients (≥ 83%) with pathognomonic LTA abnormalities from either BSS, suspected *ITGA2/ITGB3*‐RT or type 2B VWD, and the two patients with suspected MYH9RD (PLPRP: 45–59 × 10^9^ platelets/L), had been evaluated with PLPRP containing ≤ 80 × 10^9^ platelets/L, whereas most patients with some other disorders had been evaluated with PLPRP that had much higher platelet counts (e.g., those with: FPDMM, Quebec platelet disorder, *TUBB1*‐related thrombocytopenia, Jacobsen syndrome, thrombocytopenia from liver disease, or non‐diagnostic findings) (Tables 2, S2).

### 3.5 Intra‐Subject MA Variability Within Tests With Replicates for Patients and Controls

Table 3 summarizes median and ranges for intra‐subject, within‐test MA CV data, and differences in %MA estimates for replicates for informative agonists, for samples with different platelet count ranges. For both CLPRP and PLPRP, the median within‐test CVs were ≤ 18% for all agonists except 0.5 mg/mL ristocetin, across all ranges of sample platelet counts (Table 3). All individual subject's results with MA CVs ≥ 20% for 1.25 mg/mL ristocetin, 5.0 μg/mL collagen, 1.6 mM AA, and/or 1.0 μM U46619 had an abnormally reduced MA. Moreover, tests showing normal responses to these latter agonists and to 1.25 μg/mL collagen had significantly lower CVs than those with impaired response findings (*p* values < 0.01). While some PLPRP with normal or abnormal MA responses to 2.5 and 5.0 μM ADP and 1.25 μg/mL collagen had MA CVs >20%, differences between %MA estimates, within replicates, were small (range: 2%–7% for: 1.25 μg/mL collagen and 2.5 and 5.0 μM ADP) (Table 3). With 1.25 mg/mL ristocetin only, there was a weak, inverse association between MA CVs and sample platelet counts (r^2^–0.04, *p* = 0.04).

**TABLE 3:** Within‐test variability in maximal aggregation responses to informative agonists for low platelet count samples from patients and controls. Variability for samples tested at different platelet counts was assessed using coefficients of variation and the differences between % maximal aggregation estimates for replicates.

Median CV (range) for MA estimates						
Differences between %MA estimates for replicates as median [range]						
Numbers of persons/tests with ≥ 2 replicates						
Samples	Patient LPRP	Control LPRP	Patient LPRP	Control LPRP	Patient LPRP	Controls
Platelet counts	9–80 × 10^9^/L	9–80 × 10^9^/L	81–140 × 10^9^/L	81–140 × 10^9^/L	141–249 × 10^9^/L	141–249 × 10^9^/L
1.25 mg/mL ristocetin	CV 3.9 (0.0–141)%^a^ 1.5 [0–16] *n* = 34/35	CV 9.1 (2.1–24)%^a^ 9 [2–14] *n* = 5/5	CV 3.5 (0.0–200)%^a^ 3 [0–19] *n* = 39/40	CV 2.0 (1.8–2.3)% 2.5 [2–3] *n* = 2/2	CV 4.9 (0.0–51)%^a^ 5 [0–28] *n* = 40/46	CV 5.6 (4.1–7.2)% 6.5 [5–8] *n* = 2/2
0.5 mg/mL ristocetin	CV 24 (0.0–142)%^b^ 2 [0–17] *n* = 27/27	CV 37 (0.0–57)%^b^ 5 [1–7] *n* = 5/5	CV 10 (0–141)%^b^ 1 [0–12] *n* = 20/22	CV 55 (28–81)%^b^ 5 [2–8] *n* = 2/2	CV 14 (0.0–173)%^b^ 2 [0–20] *n* = 15/18	CV 44 (NA)%^b^ 5 [NA] *n* = 1/1
5 μg/mL collagen	CV 5.7 (1.1–53)%^a^ 2.5 [1–18] *n* = 22/22	CV 5.0 (4.1–9.1)% 4 [NA] *n* = 3/3	CV 3.1 (0.0–53)%^a^ 3 [0–11] *n* = 31/32	CV 2.5 (0.8–17)% 2.5 [1–8] *n* = 4/4	CV 1.9 (0.0–71)%^a^ 2 [0–10] *n* = 48/52	CV 0.7 (NA)% 1 [NA] *n* = 1/1
1.25 μg/mL collagen	NA	NA	NA	NA	CV 17 (1.1–99)%^b^ 5 [1–33] *n* = 21/21	CV 13 (6.6–18)% 16 [7–25] *n* = 2/2
5 μM ADP	NA	NA	CV 9.9 (0.0–25)%^b^ 4 [0–32] *n* = 25/27	CV 6.7 (6.4–13)% 5 [1–16] *n* = 3/4	CV 6.1 (0.0–141)%^b^ 4 [0–20] *n* = 28/29	CV 1.6 (NA)% 2 [NA] *n* = 1/1
2.5 μM ADP	NA	NA	NA	NA	CV 8.8 (0.8–26)%^b^ 2 [0–11] *n* = 18/20	CV 7.6 (2.5–13)% 7 [3–11] *n* = 2/2
1.6 mM AA	NA	NA	NA	NA	CV 3.8 (0.0–113)%^a^ 2.5 [0–10] *n* = 26/29	CV 0.6 (0.0–4.4)% 1 [0–4] *n* = 3/3
1.0 μM thromboxane analog U46619	NA	NA	NA	NA	CV 14 (0.0–141)%^a^ 5 [0–20] *n* = 45/47	CV 18 (8.2–28)%^a^ 6 [3–9] *n* = 2/2
6 μM epinephrine^c^	NA	NA	NA	NA	CV 2.3 (NA)% 3 [NA] *n* = 1/1	No data

Abbreviations: CV, coefficient of variation; MA, maximal aggregation; NA, not applicable.

^a^ CVs ≥ 20% were exclusive to replicates with abnormally reduced MA.

^b^ CV ≥ 20% were not exclusive to replicates with abnormal MA.

^c^ Epinephrine was only evaluated for samples with 240–249 × 10^9^ platelets/L.

With 0.5 mg/mL ristocetin (which induced no visible agglutination/aggregation in most samples), the median intra‐subject, within‐test MA CVs, were 10%–14% for PLPRP and 37%–55% for CLPRP across the different platelet count ranges (Table 3), with significantly lower CVs for PLPRP that showed abnormally increased vs. normal MA (*p* = 0.014). The small median differences in %MA for replicates with 0.5 mg/mL ristocetin (PLPRP: 1%–2%; CLPRP: 2%–5%) suggested acceptable precision (Table 3).

### 3.6 Variability for MA Estimates Across Tests

Between‐test agonist responses (within‐test outliers excluded) were evaluated for 41 patients across 43 sets of tests (2–6 MA determinations/agonist with replicates, *n* = 2 patients with between‐test data for two different platelet count ranges), and for 53 controls across 74 sets of tests (2–21 MA determinations/agonist with replicates, 7 with data for two different platelet count ranges, and 7 with data for three platelet count ranges, each analyzed separately). Table 4 summarizes within‐subject, between‐test MA CV data for PLPRP and CLPRP tests with informative agonists. Although MA CVs were highest for 0.5 mg/mL ristocetin across all ranges of sample platelet counts, 87% of patients and 97% of controls had consistently normal MA responses that reflected no visible agglutination/aggregation response to 0.5 mg/mL ristocetin, and the median differences between %MA estimates across tests/person were small (Table 4). The median MA CVs across tests/person were < 20% for 1.25 mg/mL ristocetin and 5 μg/mL collagen, and higher for responses to weak agonists (Table 4). Intra‐subject, between‐test PLPRP MA CVs were significantly higher for persons with impaired than normal MA responses (*p* values ≤ 0.007) to all agonists except 0.5 mg/mL ristocetin, 2.5 μM ADP, and 1.25 μg/mL collagen. For 1.25 mg/mL ristocetin only, intra‐subject between‐test MA CVs showed a positive association to the between‐test differences in PLPRP platelet counts (r2 = 0.15, *p* = 0.015; median, range, IQR for platelet count differences × 10^9^/L: 22, 0–135, 6–46).

**TABLE 4:** Between‐test variability in maximal aggregation responses to informative agonists for low platelet count samples from patients and controls. Variability across tests was assessed using coefficients of variation and the differences between maximal aggregation estimates across tests. Epinephrine data were limited (only evaluated for samples with ≥ 240 × 10^9^ platelets/L).

Median CV (range) for MA estimates						
Differences between %MA estimates across tests as median [range]						
Numbers of persons with ≥ 2 samples						
[Range of differences for platelet counts (×10^9^/L) of compared samples]						
Samples	Patient LPRP	Control LPRP	Patient LPRP	Control LPRP	Patient LPRP	Control LPRP
Platelet counts	9–80 × 10^9^/L	9–80 × 10^9^/L	81–140 × 10^9^/L	81–140 × 10^9^/L	141–249 × 10^9^/L	141–249 × 10^9^/L
1.25 mg/mL ristocetin	CV 47 (11–97)%^a^ 16 [1–50] *n* = 8	CV 12 (2.1–43)%^b^ 21 [2–40] *n* = 13	CV 15 (0–78)%^a^ 20 [0–82] *n* = 9	CV 7.1 (0.8–25)%^b^ 12 [0–32] *n* = 23	CV 4.5 (0–113)%^b^ 7 [0–79] *n* = 23	CV 5.0 (0–36)%^a^ 27 [1–61] *n* = 42
0.5 mg/ml ristocetin	CV 66 (0–141)%^b^ 1 [0–11] *n* = 7	CV 47% (0–143)^b^ 5 [0–21] *n* = 11	CV 42 (2.3–79)%^b^ 3 [1–9] *n* = 9	CV 72% (0–141)^b^ 3 [0–11] *n* = 23	CV 47 (0.0–200)%^b^ 1 [0–8] *n* = 21/22	CV 55 (0.0–173%)^b^ 2 [0–7] *n* = 42
5 μg/mL collagen	CV 31 (1.6–92)%^a^ 18 [2–73] *n* = 7	CV 17 (6.3–45)%^b^ 22 [11–54] *n* = 10	CV 11 (0–102)%^b^ 10 [0–56] *n* = 9	CV 5.6 (0–24^a^)%^b^ 9 [0–31] *n* = 22	CV 10 (0–161)%^b^ 11.5 [0–81] *n* = 24	CV 2.7 (0.7–28)%^a^ 4 [1–41] *n* = 42
1.25 μg/mL collagen	NA	NA	NA	NA	CV 32 (16–129)%^b^ 28 [14–50] *n* = 6	CV 7.8 (0–62)%^b^ 9 [0–76] *n* = 26/27
5 μM ADP	NA	NA	CV 20 (4.5–47)%^b^ 10 [3–23] *n* = 7	CV 25 (5.7–55)%^b^ 27 [1–61] *n* = 13	CV 20 (2.8–101)%^b^ 10 [3–52] *n* = 22	CV 7.1 (0.7–49)%^b^ 12 [1–56] *n* = 41
2.5 μM ADP	NA	NA	NA	NA	CV 24 (4.0–173)%^b^ 13 [0–67] *n* = 20/22	CV 24 (0.0–64)%^b^ 26 [0–78] *n* = 41
1.6 mM AA	NA	NA	NA	NA	CV 18 (0.8–108)%^b^ 19 [1–73] *n* = 16/17	CV 4.3 (0.0–43)%^b^ 7 [0–54] *n* = 41
1.0 μM thromboxane analog U46619	NA	NA	NA	NA	CV 35 (1.6–121)%^b^ 10 [1–78] *n* = 19	CV 3.4 (0–94)%^b^ 7 [0–76] *n* = 39
6 μM epinephrine	NA	NA	NA	NA	No data	CV 3.6 (3.0–4.7)% 7 [4–8] *n* = 3

Abbreviations: CV, coefficient of variation; MA, maximal aggregation; NA, not applicable.

^a^ CVs ≥ 20% were exclusive to persons that had reduced MA on ≥ 1 estimate.

^b^ CV ≥ 20% were not exclusive to persons with abnormal MA findings.

### 3.7 Agreement Between Aggregation Tests Showing Normal or Abnormal Findings

Table 5 summarizes the intra‐subject, between‐test agreement for whether MA responses to individual agonists and overall test findings were consistent, using data for 53 controls and 41 patients. Most controls and patients had consistent MA findings with individual agonists across tests, with significantly more patients than controls having consistently abnormal MA findings with 1.25 mg/mL ristocetin, 5 μg/mL collagen, 5 μM ADP, and 1 μM U46619 (*p* values ≤ 0.02; Table 5). Most controls (98.1%) had overall consistent, normal/non‐diagnostic findings across all tests (reduced MA with U46619 only was classified as a non‐diagnostic finding) (Table 5, Table S3). Most patients had overall consistent LTA findings across all tests (80.5%), with 46.3% having consistent, abnormal findings, 34.1% having consistent normal findings, and 19.5% having variable findings (Table 5, Table S2). All patients with pathognomonic aggregation abnormalities (e.g., from BSS, type 2B VWD, and suspected *ITGA2/ITGB3*‐RT) had consistent, abnormal findings, as did patients with FPDMM and PFD secondary to underlying hematological disorders and some other disorders (details in Table 5 footnote). Patients with inconsistent LTA findings had a variety of diagnoses, including conditions that resolved (e.g., transient acquired thrombocytopenia, transient acquired BSS, ITP, and NSAID‐induced platelet dysfunction; details in Table 5 footnote).

**TABLE 5:** Between‐test agreement in maximal aggregation responses for agonists and overall findings for low platelet count platelet‐rich plasma. Maximal aggregation data compares data for ≥ 2 tests for unique samples, with findings for LPRP with ≤ 80, 81–140 or 141–249 × 10^9^ platelets/L treated as separate comparisons as recommended agonists differ. Maximal aggregation findings for an agonist were excluded if there were < 2 separate test results. Some controls and patients had maximal aggregation data for 2 or 3 platelet count ranges (overall findings considered all of their test results). *p* values indicate significantly more abnormalities for patients than controls.

		% (proportion) with replicates considered, *p* values indicate significantly more abnormal or variable results than corresponding controls			
Group, parameter evaluated	Parameter evaluated	Consistent (normal or abnormal)	Consistently normal (or non‐diagnostic)	Consistently abnormal	Variable (normal/abnormal)
Controls MA	1.25 mg/mL ristocetin	92% (68/74)	92% (68/74)	0%	8.1% (6/74)
	0.5 mg/mL ristocetin	97% (70/72)	97% (70/72)	0%	3% (2/72)
	5 μg/mL collagen	94% (30/32)	91% (29/32)	3% (1/32)	6% (2/32)^a^
	1.25 μg/mL collagen	96% (22/23)	96% (22/23)	0%	4% (1/23)
	5 μM ADP	98% (49/50)	98% (49/50)	0%	2% (1/50)^a^
	2.5 μM ADP	100% (37/37)	100% (37/37)	0%	0%
	1.6 mm AA	100% (37/37)	100% (37/37)	0%	0%
	1.0 μM U46610	94% (33/35)	94% (33/35)	0%	6% (2/35)
Controls, Overall LTA interpretation	Entire panel, all tests	98.1% (52/53)	98.1% (52/53)	0%	1.9% (1/53)^a^
Patients MA	1.25 mg/mL ristocetin	75% (30/40)	50% (20/40)	25% (10/40) *p* < 0.001	25% (10/40)
	0.5 mg/mL ristocetin	97% (36/37)	95% (35/37)	2.7% (1/37)	5.4% (1/37)
	5 μg/mL collagen	78% (31/40)	55% (22/40)	22.5% (9/40) *p* = 0.02	22.5% (9/40)
	1.25 μg/mL collagen	67% (4/6)	50% (3/6)	17% (1/6)	33% (2/6)
	5 μM ADP	97% (28/29)	69% (20/29)	28% (8/29) *p* < 0.001	3% (1/29)
	2.5 μM ADP	95% (19/20)	85% (17/20)	10% (2/20)	5% (1/20)
	1.6 mm AA	88% (15/17)	71% (12/17)	18% (3/17)	12% (2/17)
	1.0 μM U46619	74% (14/19)	32% (6/19)	42% (8/19) *p* < 0.001	26% (5/19)
Patients, Overall LTA interpretation	Entire panel, all tests	80.5% (32/41)	34.1% (13/41)^b^	(19/41)^c^ *p* < 0.001	19.5% (9/41)^d^

Abbreviations: LTA, light transmittance aggregometry; MA, maximal aggregation.

^a^ One control had mildly reduced MA, considered to be a false positive, with 5 μg/mL collagen and 5 μM ADP on one of two tests for LPRP with 81–140 × 10^9^ platelets/L.

^b^ Diagnoses for patients with consistently normal/non‐diagnostic findings included: thrombocytopenia of unknown cause, including familial (*n* = 7), Jacobsen syndrome (*n* = 1), thrombocytopenia secondary to non‐hematological malignancy (*n* = 1), bleeding problems of unknown cause (*n* = 1), and *n* = 2 unknown (external patients).

^c^ Diagnoses for patients with consistently abnormal LTA findings included: platelet function disorders of unknown cause (*n* = 5, one with a *RUNX1* variant of uncertain significance, one with dense granule deficiency), thrombocytopenia of unknown cause, including familial (*n* = 4, one with dense granule deficiency), Bernard–Soulier syndrome (*n* = 3), type 2B von Willebrand disease (*n* = 1), suspected *ITGA2/ITGB3*‐related thrombocytopenia (*n* = 1), familial platelet function disorders with predisposition to myeloid malignancy (*n* = 1), acquired platelet function disorders complicating myelodysplasia (*n* = 1), chronic lymphocytic leukemia (*n* = 1), liver disease (*n* = 1), or immune thrombocytopenia (*n* = 1, with reduced ristocetin‐induced platelet aggregation).

^d^ Diagnoses for patients with variable LTA findings included: familial thrombocytopenia of unknown cause (*n* = 4; one had non‐steroidal anti‐inflammatory drug interference on one test), antiphospholipid antibody syndrome (*n* = 1), transient acquired Bernard–Soulier syndrome (*n* = 1), immune thrombocytopenia (*n* = 1), transient thrombocytopenia (*n* = 1), and *n* = 1 unknown (external patient).

## 4 Discussion

Our major goal was to assess the trustworthiness of LTA with low platelet samples, using data for QA samples, and retrospective analysis of “real‐life” diagnostic samples with replicates, to assess test endpoint precision and the reproducibility of agonist responses and overall findings. The retrospective component that evaluated a large number of consecutive patients (*n* = 195 of diverse ages and diagnoses) increases the likelihood of external validity for our estimates of the “real‐life” precision and reproducibility of LPRP LTA for “ruling‐out” and “ruling‐in” inherited and acquired PFD of diverse causes.

Concerns have been raised that some very low platelet count samples are not suitable to assess platelet function [13]. Our QA data for CLPRP, with 8 replicates/agonist/sample, indicated MA precision was poorer for samples with ≤ 25 × 10^9^ platelets/L (Table 1, Table S1), which had more difficult‐to‐interpret LTA tracings (Figure S1). However, only 0%–1.9% of consecutive PLPRP have ≤ 25 × 10^9^ platelets/L [1, 2]. Moreover, we found that five out of seven PLPRP tested at ≤ 25 × 10^9^/L had reportable, pathognomonic BSS findings (four with congenital BSS, one with ITP and acquired BSS). Due to macrothrombocytopenia, BSS samples are particularly prone to poor platelet recovery, particularly if centrifuged (a detail that we now document). Automated cell counters can underestimate BSS platelet counts and platelet size [22], which could be why pathognomonic BSS LTA findings were easily identified among samples with ≤ 25 × 10^9^ platelets/L (Figure S2) and higher platelet counts. It remains challenging to identify samples requiring gravity separation in advance, given the inaccuracies in estimating platelet counts and size for macrothrombocytopenic disorders [22].

Among the CLPRP and PLPRP that we examined, MA outliers were infrequent (Tables S2, S3) and MA precision for informative agonists was acceptable across a wide range of LPRP platelet counts (including those ≤ 80 × 10^9^ platelets/L). Our estimates of intra‐subject CLPRP MA CVs for agonists are quite similar to those of normal platelet count samples [18, 19]. MA CVs were higher for 0.5 mg/mL ristocetin, weak agonists, impaired aggregation responses (Table 3), and across‐test MA estimates (Table 4); however, absolute differences between MA estimates for these responses were small and indicative of adequate precision (Table 3). Importantly, LPRP MA responses, and overall test findings, were consistent across tests for most patients and controls (Table 5). Taken together, these data indicate that LPRP LTA has acceptable precision and good reproducibility when performed by standard procedures with informative agonists.

Our study has some inherent weaknesses. As we used one manufacturer's aggregometers to test LPRP (PACKS‐4 and AggRAM), our key findings need validation for other instruments. The CLPRP QA data had more replicates (8/agonist) to estimate MA CVs than the retrospective CLPRP and PLPRP data (2–4/agonist). The retrospective component likely introduced some bias, as there were more MA estimates and more repeat tests when PLPRP showed abnormal compared to non‐diagnostic findings. Nonetheless, quite a few historical PLPRP had within‐test MA replicates for normal agonist responses, possibly because laboratory technologists wanted to ensure results were reportable, given the limited number of informative agonists for LPRP with ≤ 140 × 10^9^ platelets/L. Furthermore, laboratory technologists may have been uncertain about which findings were abnormal, particularly before LPRP MA RI were established [1].

An earlier study by Quiroga and colleagues [19] established most patients with inherited mucocutaneous bleeding problems, tested by LTA with PRP containing 250 × 10^9^ platelets/L, have consistent findings, with 40% being consistently abnormal and suggestive of a PFD. Our study of LPRP had a slightly higher percentage of patients with consistently abnormal (46.3% vs. 40%) or variable findings (19.5% vs. 8%) across tests, which likely reflect differences in patient populations, study design, definitions of abnormal findings, PRP platelet counts, and test procedures. Like Quiroga [19], we found that most patients with normal findings had confirmed normal findings on follow‐up tests. Importantly, in our cohort, patients with pathognomonic LTA abnormalities had consistently abnormal findings, as did many patients with other PFD, whereas those who had drug interference or conditions that improved, had variable findings.

We conclude that LTA assessment of LPRP has acceptable precision and reproducibility and recommend:

1. Gravity separation of PRP for patients with known or suspected macrothrombocytopenic disorders with a cautious examination for BSS findings or abnormalities with multiple agonists, suggestive of *ITGA2/ITGB3*‐RT or ITP with acquired GT (with or without acquired BSS) [2] if platelet recovery is very poor (e.g., ≤ 25 × 10^9^ platelets/L PRP, or the lowest count, locally validated to give interpretable findings with ristocetin and a high concentration of collagen, such as 5 μg/mL Horm collagen).
2. If sample volumes permit, do additional MA replicates for LPRP with ≤ 50 × 10^9^ platelets/L, and/or impaired MA responses, to facilitate test interpretation.
3. Confirm PLPRP LTA abnormalities with multiple agonists and/or ristocetin on a second sample, unless the suspected diagnosis can be confirmed by other methods (e.g., flow cytometry and/or genetic analysis to verify congenital BSS, *ITGA2/ITGB3*‐RT, or type 2B VWD).

## Author Contributions

C.P.M.H. obtained research ethics approval, reviewed clinical aggregation test records to obtain data on replicates for low platelet count samples, performed electronic medical record reviews to obtain information on diagnoses and relevant laboratory findings, supervised the study, and led the manuscript preparation and preparation of the supplementary information file, with contributions from all the authors. L.W. tested and analyzed QA samples. R.A.D. led statistical analysis. K.A.M. analyzed historical data for patient and control sample replicates.

## Conflicts of Interest

The authors declare no conflicts of interest.

## Supporting information


Data S1.

## Data Availability

The data that supports the findings of this study are available in the supplementary material of this article.

## References

[1] C. P. Hayward , K. A. Moffat , M. Pai , et al., “An Evaluation of Methods for Determining Reference Intervals for Light Transmission Platelet Aggregation Tests on Samples With Normal or Reduced Platelet Counts,” Thrombosis and Haemostasis 100, no. 1 (2008): 134–145. 18612548

[2] R. M. Altahan , N. Mathews , A. Bourguignon , et al., “Evaluation of a Diagnostic Platelet Aggregation Test Strategy for Platelet Rich Plasma Samples With Low Platelet Counts,” International Journal of Laboratory Hematology 46, no. 2 (2024): 362–374. 38148642 10.1111/ijlh.14216

[3] A. Bourguignon , S. Tasneem , and C. P. Hayward , “Screening and Diagnosis of Inherited Platelet Disorders,” Critical Reviews in Clinical Laboratory Sciences 59, no. 6 (2022): 405–444. 35341454 10.1080/10408363.2022.2049199

[4] P. Gresele and Subcommittee on Platelet Physiology of the International Society on T, Hemostasis , “Diagnosis of Inherited Platelet Function Disorders: Guidance From the SSC of the ISTH,” Journal of Thrombosis and Haemostasis 13, no. 2 (2015): 314–322. 25403439 10.1111/jth.12792

[5] W. L. Nichols , S. E. Kaese , D. A. Gastineau , L. A. Otteman , and E. J. Bowie , “Bernard‐Soulier Syndrome: Whole Blood Diagnostic Assays of Platelets,” Mayo Clinic Proceedings 64, no. 5 (1989): 522–530. 2725065 10.1016/s0025-6196(12)65556-6

[6] C. Lacom , A. Tolios , M. W. Loffler , et al., “Assay Validity of Point‐Of‐Care Platelet Function Tests in Thrombocytopenic Blood Samples,” Biochemia Medica 32, no. 2 (2022): 020713. 35799989 10.11613/BM.2022.020713 PMC9195599

[7] M. T. Skipper , P. Rubak , J. Stentoft , A. M. Hvas , and O. H. Larsen , “Evaluation of Platelet Function in Thrombocytopenia,” Platelets 29, no. 3 (2018): 270–276. 28409645 10.1080/09537104.2017.1296566

[8] A. A. Hanke , K. Roberg , E. Monaca , et al., “Impact of Platelet Count on Results Obtained From Multiple Electrode Platelet Aggregometry (Multiplate),” European Journal of Medical Research 15, no. 5 (2010): 214–219. 20562061 10.1186/2047-783X-15-5-214 PMC3352011

[9] T. Stissing , N. P. Dridi , S. R. Ostrowski , L. Bochsen , and P. I. Johansson , “The Influence of Low Platelet Count on Whole Blood Aggregometry Assessed by Multiplate,” Clinical and Applied Thrombosis/Hemostasis 17, no. 6 (2011): E211–E217. 21406416 10.1177/1076029610397183

[10] M. Wurtz , A. M. Hvas , S. D. Kristensen , and E. L. Grove , “Platelet Aggregation Is Dependent on Platelet Count in Patients With Coronary Artery Disease,” Thrombosis Research 129, no. 1 (2012): 56–61. 21917303 10.1016/j.thromres.2011.08.019

[11] M. Tiedemann Skipper , P. Rubak , O. Halfdan Larsen , and A. M. Hvas , “Thrombocytopenia Model With Minimal Manipulation of Blood Cells Allowing Whole Blood Assessment of Platelet Function,” Platelets 27, no. 4 (2016): 295–300. 26555800 10.3109/09537104.2015.1095873

[12] G. Kuiper , R. Houben , R. J. H. Wetzels , et al., “The Use of Regression Analysis in Determining Reference Intervals for Low Hematocrit and Thrombocyte Count in Multiple Electrode Aggregometry and Platelet Function Analyzer 100 Testing of Platelet Function,” Platelets 28, no. 7 (2017): 668–675. 28067094 10.1080/09537104.2016.1257782

[13] N. Boknas , A. S. Macwan , A. L. Sodergren , and S. Ramstrom , “Platelet Function Testing at Low Platelet Counts: When Can You Trust Your Analysis?,” Research and Practice in Thrombosis and Haemostasis 3, no. 2 (2019): 285–290. 31011713 10.1002/rth2.12193 PMC6462761

[14] E. A. Femia , M. Scavone , A. Lecchi , and M. Cattaneo , “Effect of Platelet Count on Platelet Aggregation Measured With Impedance Aggregometry (Multiplate Analyzer) and With Light Transmission Aggregometry,” Journal of Thrombosis and Haemostasis 11, no. 12 (2013): 2193–2196. 24148217 10.1111/jth.12432

[15] A. S. Lawrie , K. Kobayashi , P. J. Lane , I. J. Mackie , and S. J. Machin , “The Automation of Routine Light Transmission Platelet Aggregation,” International Journal of Laboratory Hematology 36, no. 4 (2014): 431–438. 24237750 10.1111/ijlh.12161 PMC4298021

[16] P. J. Vinholt , H. Frederiksen , A. M. Hvas , U. Sprogoe , and C. Nielsen , “Measurement of Platelet Aggregation, Independently of Patient Platelet Count: A Flow‐Cytometric Approach,” Journal of Thrombosis and Haemostasis 15, no. 6 (2017): 1191–1202. 28296243 10.1111/jth.13675

[17] P. J. Vinholt , A. M. Hvas , and M. Nybo , “An Overview of Platelet Indices and Methods for Evaluating Platelet Function in Thrombocytopenic Patients,” European Journal of Haematology 92, no. 5 (2014): 367–376. 24400878 10.1111/ejh.12262

[18] C. P. M. Hayward , K. A. Moffat , J. Brunet , et al., “Update on Diagnostic Testing for Platelet Function Disorders: What Is Practical and Useful?,” International Journal of Laboratory Hematology 41, no. Suppl 1 (2019): 26–32. 31069975 10.1111/ijlh.12995

[19] T. Quiroga , M. Goycoolea , V. Matus , et al., “Diagnosis of Mild Platelet Function Disorders. Reliability and Usefulness of Light Transmission Platelet Aggregation and Serotonin Secretion Assays,” British Journal of Haematology 147, no. 5 (2009): 729–736. 19775303 10.1111/j.1365-2141.2009.07890.x

[20] “510(k) Substantial Equivalence Determination Decision Summary Instrument Only Template to Obtain Clearance for the HemoRAM System With the AggRAM Aggregation (Remote Analyzer Module) System”, https://www.accessdata.fda.gov/cdrh_docs/reviews/K050053.pdf. 2025.

[21] C. P. Hayward , K. A. Moffat , J. F. Castilloux , et al., “Simultaneous Measurement of Adenosine Triphosphate Release and Aggregation Potentiates Human Platelet Aggregation Responses for Some Subjects, Including Persons With Quebec Platelet Disorder,” Thrombosis and Haemostasis 107, no. 4 (2012): 726–734. 22234747 10.1160/TH11-10-0740

[22] P. Noris , C. Klersy , P. Gresele , et al., “Platelet Size for Distinguishing Between Inherited Thrombocytopenias and Immune Thrombocytopenia: A Multicentric, Real Life Study,” British Journal of Haematology 162, no. 1 (2013): 112–119. 23617394 10.1111/bjh.12349 PMC3757308

