# Supplementary material for: How Trustworthy is Light Transmittance Platelet Aggregometry With Low Platelet Count Samples? Insights From Test Replicates and Retrospective Analysis of Several Decades of Diagnostic Samples
Source: Int J Lab Hematol. 2025 Jun 23;47(6):1147–57. doi: 10.1111/ijlh.14518 (PMC12597866; doi:10.1111/ijlh.14518)
Supplement: Supplementary file 1 — Data S1. [file IJLH-47-1147-s001.pdf]

Supplementary Table 1: Quality assurance data generated to assess the within-test precision of light transmittance platelet aggregometry estimates for maximal aggregation. The table summarizes replicates for maximal aggregation estimates (%MA) for four unique controls, each tested with 0.5 and 1.25 mg/mL ristocetin and 5 μg/mL Horm collagen.

PRP platelet count X 10^9/L	Control 1								Control 2								Control 3								Control 4							
**5 μg/mL Collagen (%MA)**																																
**250**	96	93	96	97	94	95	93	95	87	93	88	90	91	92	92	92	94	95	97	96	95	95	96	95								
**75**	84	84	84	78	77	82	82	87	63	63	64	59	58	62	63	61	84	89	91	86	86	83	89	87								
**50**	71	70	72	74	75	74	74	71	44	43	40	42	39	36	39	41	75	77	81	76	74	76	75	76								
**25**	36	34	37	36	34	33	38	39	9	50	9	19	24	5	21	18	62	48	54	54	53	53	54	55	47	44	50	47	39	40	44	43
**20**	21	23	28	36	34	13	27	31	14	12	54	14	11	10	16	30	39	40	43	39	49	40	53	40								
**15**	51	16	42	35	22	17	19	40	14	11	27	28	16	19	28	18	20	27	23	23	37	24	20	18								
**10**	81	61	44	37	21	12	16	16	18	16	28	29	36	34	41	50	18	15	13	13	23	20	25	17								
**1.25 mg/mL Ristocetin (%MA)**																																
**250**	91	83	86	86	84	91	94	76	88	87	79	85	81	77	87	85	83	86	85	85	82	82	80	82								
**75**	79	82	84	82	83	73	75	79	74	75	75	83	77	78	77	77	89	84	84	85	87	83	82	82								
**50**	69	68	70	68	75	72	69	73	68	62	67	67	72	67	65	68	65	71	87	73	74	47	74	73								
**25**	64	71	64	61	59	62	62	58	43	41	41	36	46	49	46	42	51	49	53	60	58	48	62	45	51	52	44	55	42	44	50	42
**20**	39	31	34	30	38	40	25	41	25	35	32	30	30	29	41	30	36	39	39	91	46	76	51	34								
**15**	46	29	47	43	25	35	37	30	11	19	21	14	29	38	43	43	14	7	10	14	10	8	9	13								
**10**	39	12	33	43	35	29	11	19	10	19	14	6	24	7	10	23	10	11	16	15	12	11	6	*								
**0.5 mg/mL Ristocetin (%MA)**																																
**250**	3	1	2	1	4	2	3	4	7	3	2	3	2	5	7	2	3	2	5	2	5	1	2	2								
**75**	7	2	1	5	3	1	3	2	9	8	2	4	3	4	7	3	4	1	2	2	4	1	2	2								
**50**	6	4	6	8	2	7	4	3	8	7	6	7	9	8	4	9	1	1	3	2	7	17	6	5								
**25**	9	13	13	9	5	4	11	3	24	25	15	32	17	27	12	*	7	3	8	9	6	5	2	6	4	3	3	1	4	9	3	4
**20**	14	4	20	7	17	23	3	15	7	10	16	8	25	9	7	2	13	7	10	18	13	11	6	5								
**15**	32	21	3	14	9	32	15	*	39	17	43	9	34	17	19	*	14	24	15	11	20	23	*	*								
**10**	17	11	46	18	20	11	41	*	32	31	35	53	34	28	19	*	31	23	15	27	13	8	21	18								

* indicates insufficient sample to complete additional within-test replicates

Figure S1. The effects of control platelet rich plasma sample platelet counts on light transmittance platelet aggregometry responses to the informative agonists 5 μg/mL collagen, 1.25 mg/mL ristocetin and 0.5 mg/mL ristocetin. Panels show representative, intra-subject tracings (8 replicates/agonist/image) for platelet rich plasma adjusted to 10, 15, 20, 25, 50, 75 and 250 X 10^9 platelets/L by addition of autologous plasma. Tracings for samples with < 20 X 10^9 platelets/L were considered difficult to interpret.

5 μg/mL collagen, 250 X 10^9 platelets/L

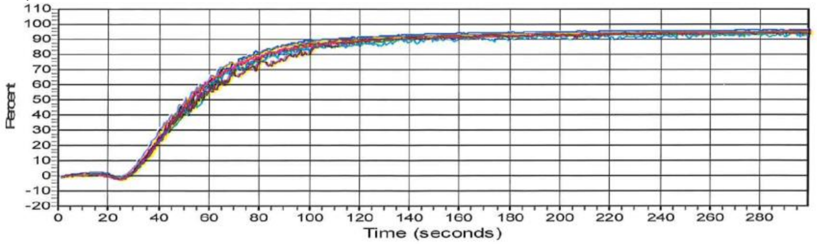

5 μg/mL collagen, 75 X 10^9 platelets/L

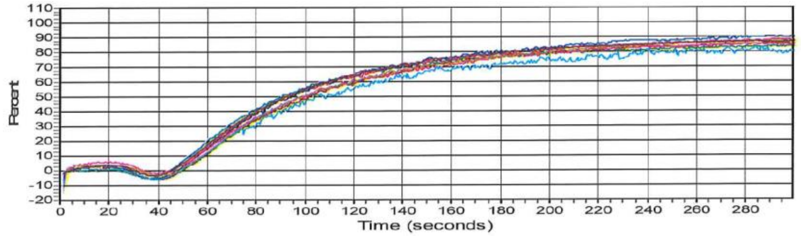

5 μg/mL collagen, 50 X 10^9 platelets/L

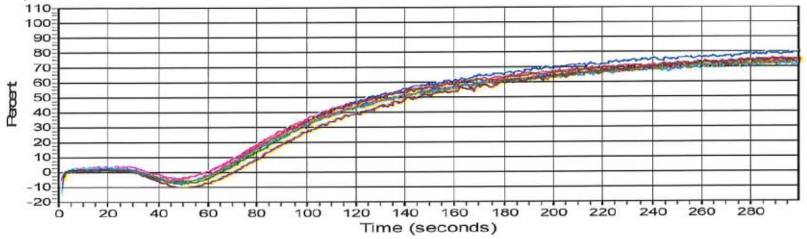

5 μg/mL collagen, 25 X 10^9 platelets/L

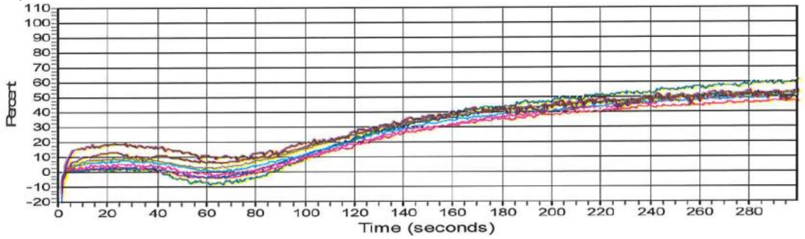

5 μg/mL collagen, 20 X 10^9 platelets/L

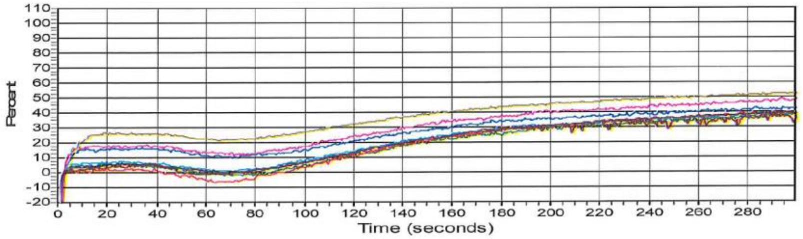

5 μg/mL collagen, 15 X 10^9 platelets/L

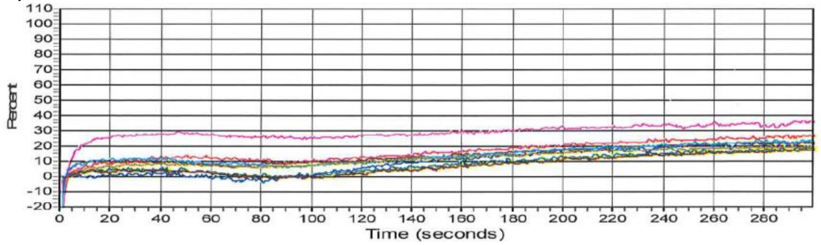

5 μg/mL collagen, 10 X 10^9 platelets/L

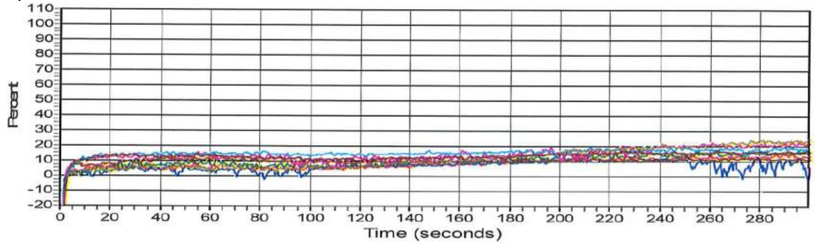

1.25 mg/mL ristocetin, 250 X 10^9 platelets/L

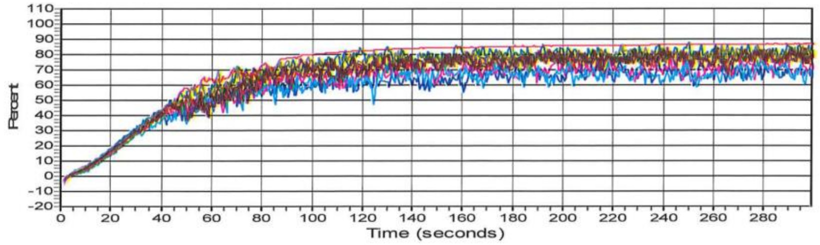

1.25 mg/mL ristocetin, 75 X 10^9 platelets/L

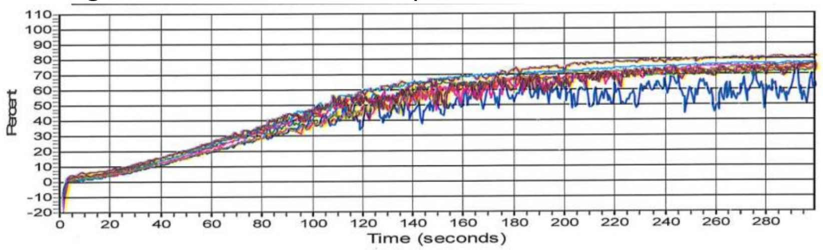

1.25 mg/mL ristocetin, 50 X 10^9 platelets/L

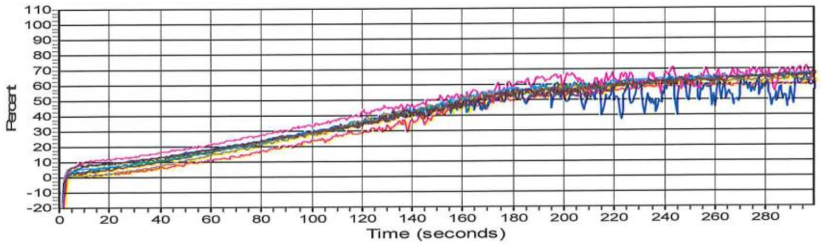

1.25 mg/mL ristocetin, 25 X 10^9 platelets/L

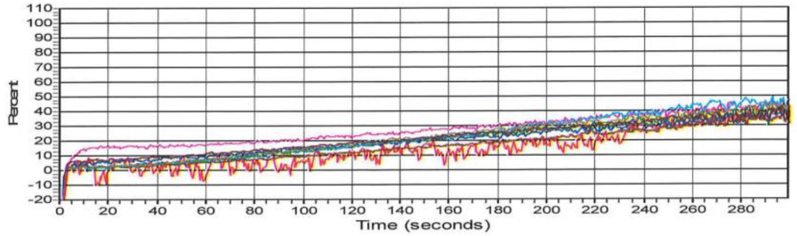

1.25 mg/mL ristocetin, 20 X 10^9 platelets/L

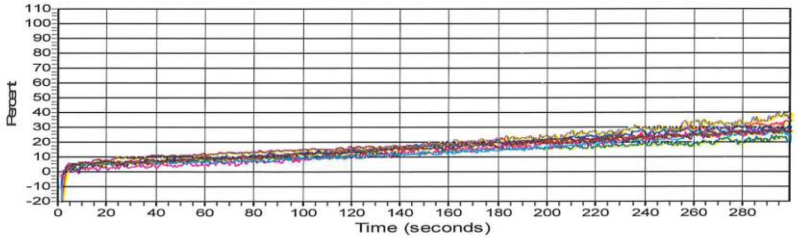

1.25 mg/mL ristocetin, 15 X 10^9 platelets/L

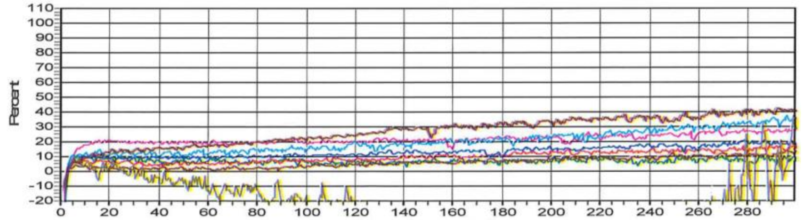

1.25 mg/mL ristocetin, 10 X 10^9 platelets/L

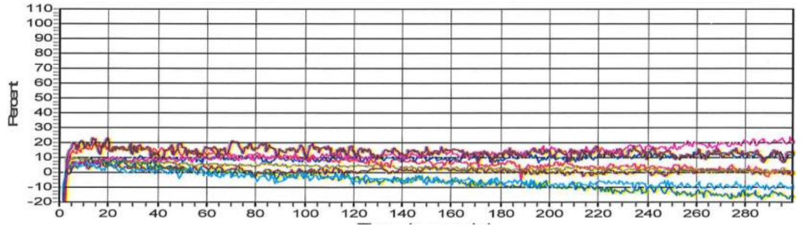

0.5 mg/mL ristocetin, 250 X 10^9 platelets/L

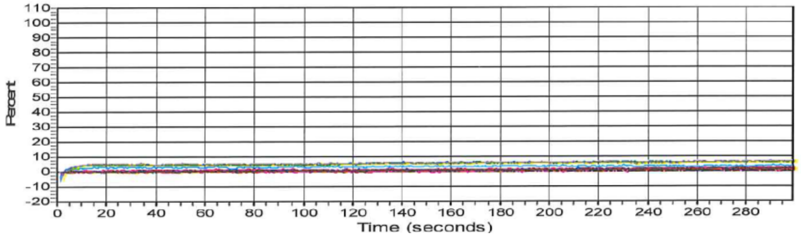

0.5 mg/mL ristocetin, 75 X 10^9 platelets/L

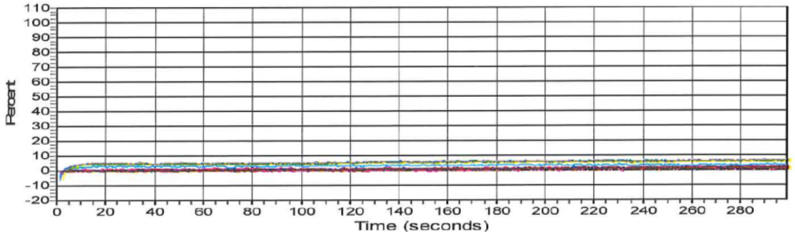

0.5 mg/mL ristocetin, 50 X 10^9 platelets/L

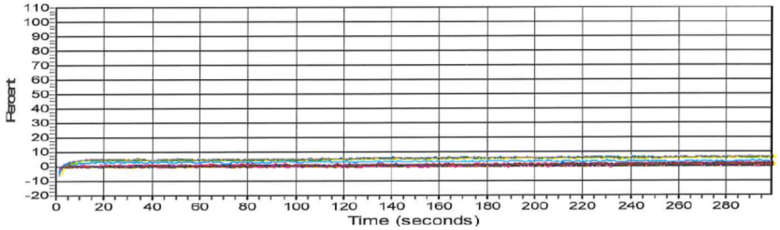

0.5 mg/mL ristocetin, 25 X 10^9 platelets/L

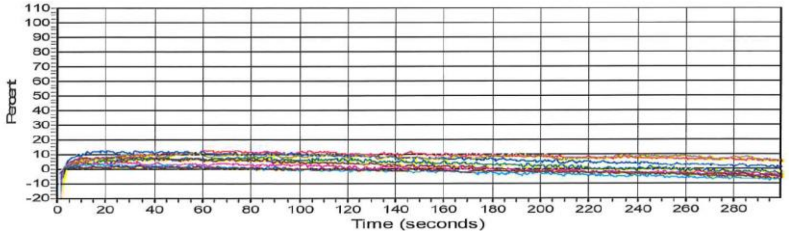

0.5 mg/mL ristocetin, 20 X 10^9 platelets/L

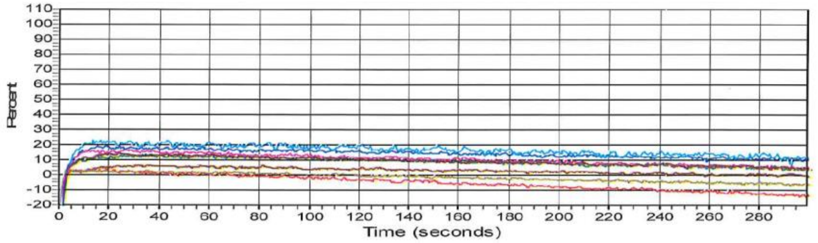

0.5 mg/mL ristocetin, 15 X 10^9 platelets/L

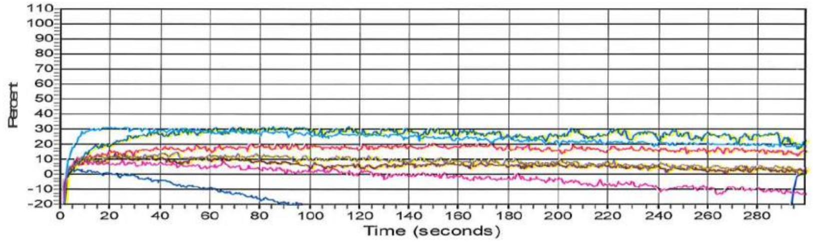

0.5 mg/mL ristocetin, 10 X 10^9 platelets/L

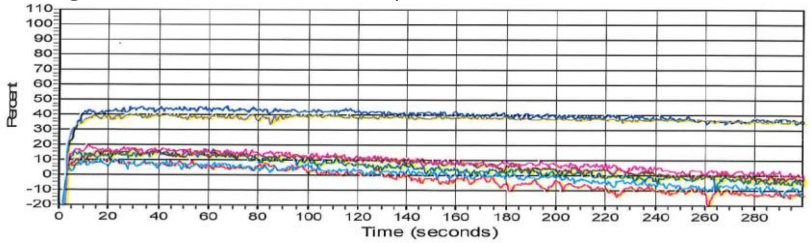

Figure S2. Aggregation tracings for the five patients (4 with inherited Bernard Soulier syndrome, one with immune thrombocytopenia complicated by acquired Bernard Soulier syndrome) who had aggregation responses evaluated for platelet rich plasma containing <25 X 10^9 platelets/L. Images illustrate that all had pathognomonic, absent agglutination/aggregation with ristocetin of congenital and acquired Bernard Soulier syndrome. Colors are indicated for the agonist responses of tested samples in each panel. PLPRP and CLPRP respectively indicate patient and control low platelet count platelet rich plasma samples. MA, maximal aggregation.

A. LTA tracing for inherited Bernard Soulier syndrome case TCP078, with PLPRP containing 9 X 10^9 platelets/L, and the corresponding control LPRP. The MA responses for the patient vs. control were: 1.25 mg/ml ristocetin: 17% (green) vs. 64% (blue); 0.5 mg/mL ristocetin: 7% (red) vs. 11% (gray). With 1.25 mg/mL ristocetin, the patient's 17% MA reflected baseline drift and "chatter." This test been rejected but was retrospectively considered reportable.

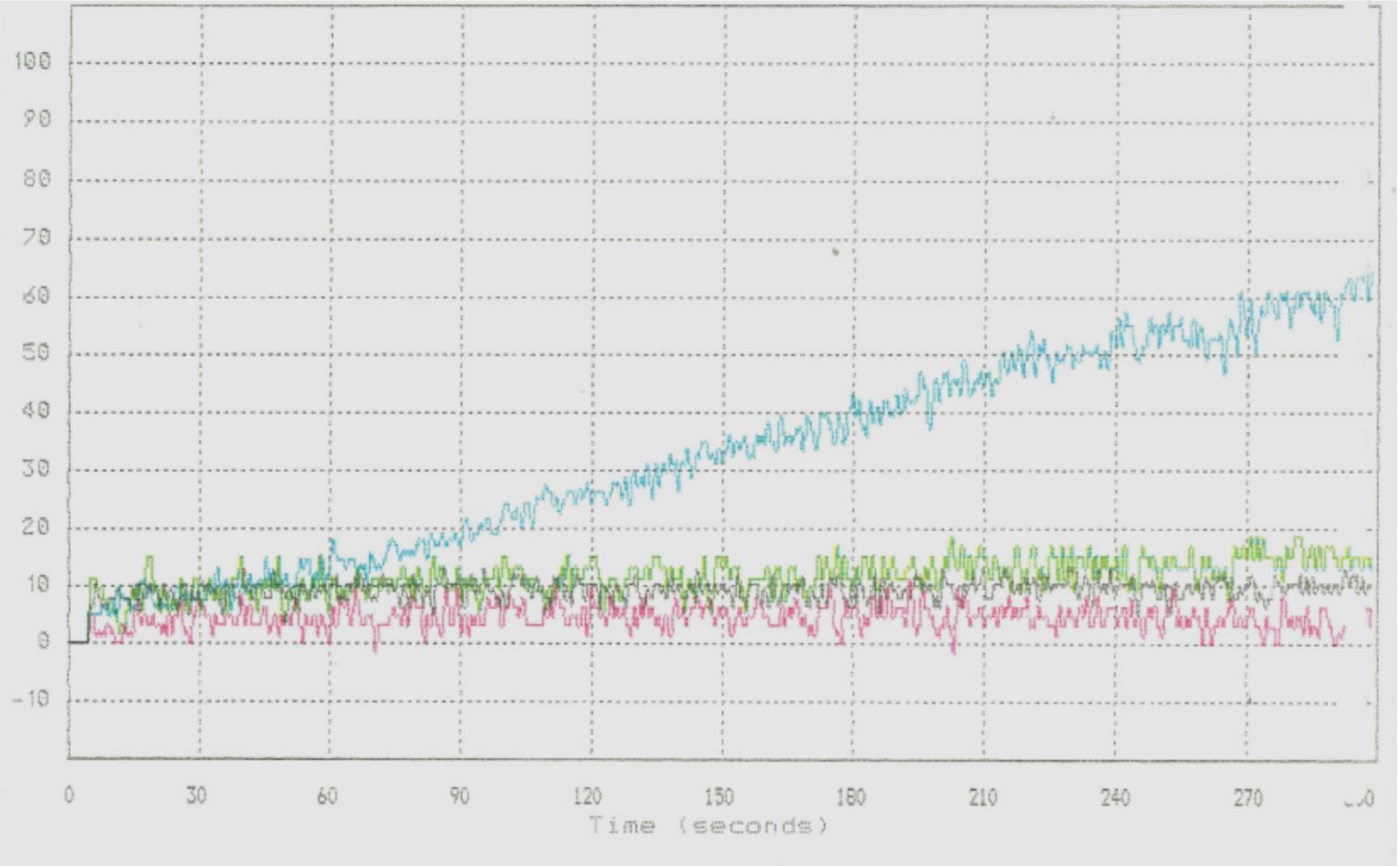

B. LTA findings for case TCP088, with immune thrombocytopenia complicated by acquired Bernard Soulier syndrome, first tested with PLPRP containing 10 X 10^9 platelets/L and compared to the CLPRP in the image below. The MA responses for the patient vs. control samples were: 1.25 mg/ml ristocetin: 1% (green) vs. 64% (blue); 0.5 mg/mL ristocetin: 1% (red) vs 4% (gray).

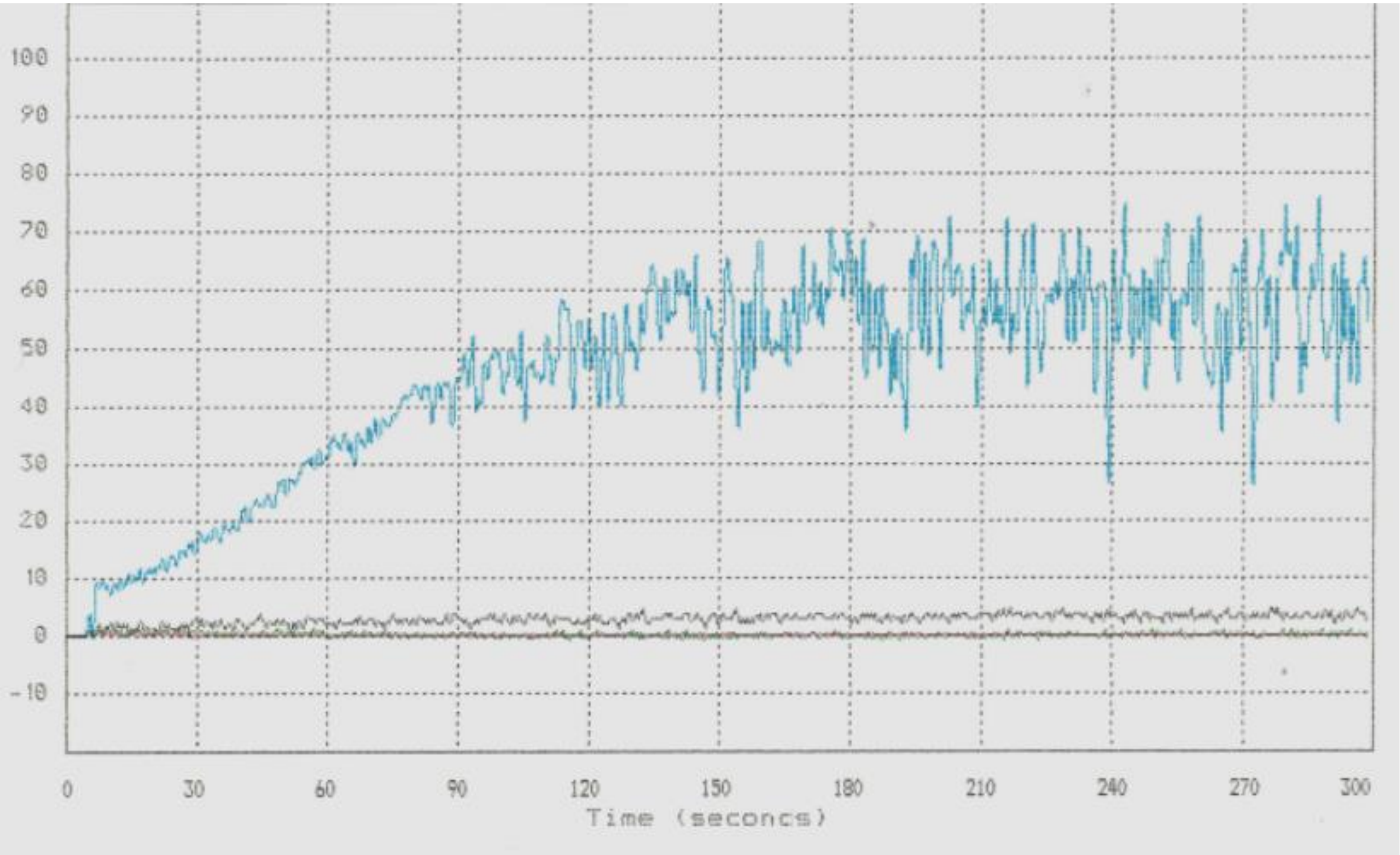

C. LTA findings for inherited Bernard Soulier syndrome case P41149, tested with PLPRP containing 17 X 10^9 platelets/L. The tracing shows the patient's platelets had an aggregation response to collagen but no visible response to ristocetin. The MA responses were: 1.25 mg/ml ristocetin: 0% (brown, channel 4); 0.5 mg/mL ristocetin: 1% (blue, channel 3); 5 µg/mL collagen: 33% (red, channel 2) and 1.25 mg/mL collagen: 33% (green, channel 1).

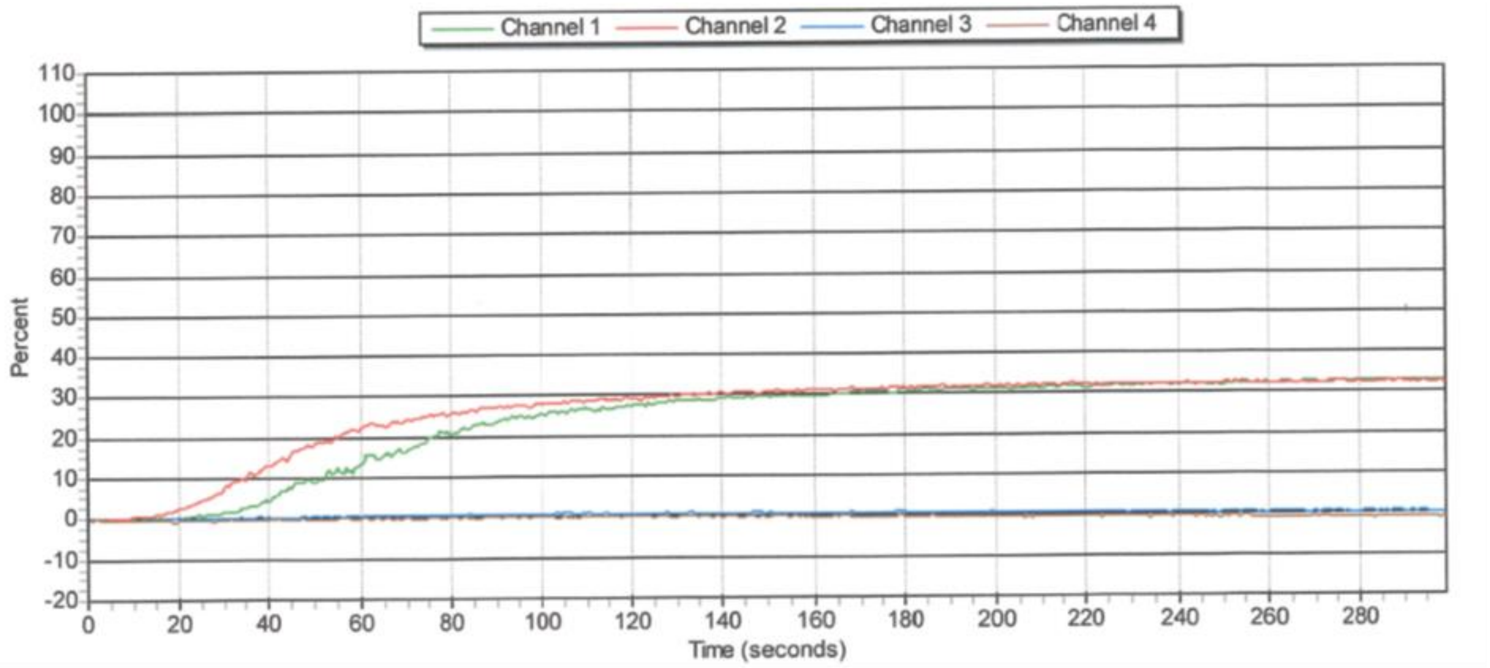

D. LTA findings for inherited Bernard Soulier syndrome case P10103, tested with PLPRP containing 19 X 10^9 platelets/L, and the simultaneously tested CLPRP. The MA responses for the patient vs. control were: 1.25 mg/ml ristocetin: 0% (red, channel 2) vs. 44% (aqua, channel 6); 0.5 mg/mL ristocetin: 1% (green, channel 1) vs 3% (pink, with downward baseline drift apparent, channel 5); 5 μg/mL collagen: 30% (blue, channel 3) vs. 32% (khaki/gray, channel 7).

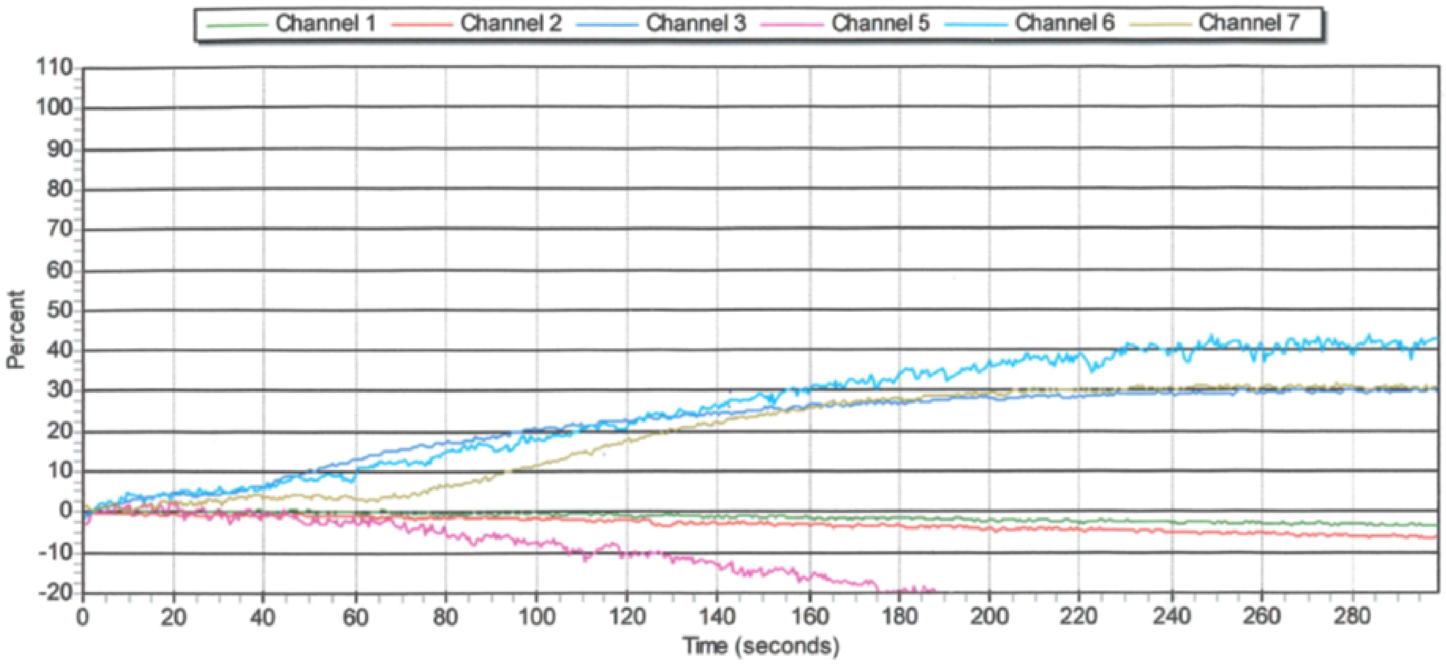

E. LTA findings for inherited Bernard Soulier syndrome case P20623, tested with PLPRP containing 19 X 10^9 platelets/L. The testing was done before policies were developed to test fewer agonists with very low platelet count samples. The patient's platelet had no agglutination response to ristocetin, with MA values of: 1.25 mg/ml ristocetin: 0% (deep magenta, channel 8); 0.5 mg/mL ristocetin: 1% (khaki gray, channel 7). However, aggregation responses were evident with all other agonists, with MA values of: 5 μg/mL collagen: 87% (brown, channel 4); 1.25 μg/mL collagen: 89% (blue, channel 3); 5 μM ADP: 87% (red, channel 2); 2.5 μM ADP: 89% (green, channel 1); 1.6 mM AA: 89% (fuchsia, channel 5); 1 μM U46619: 84% (aqua, channel 6).

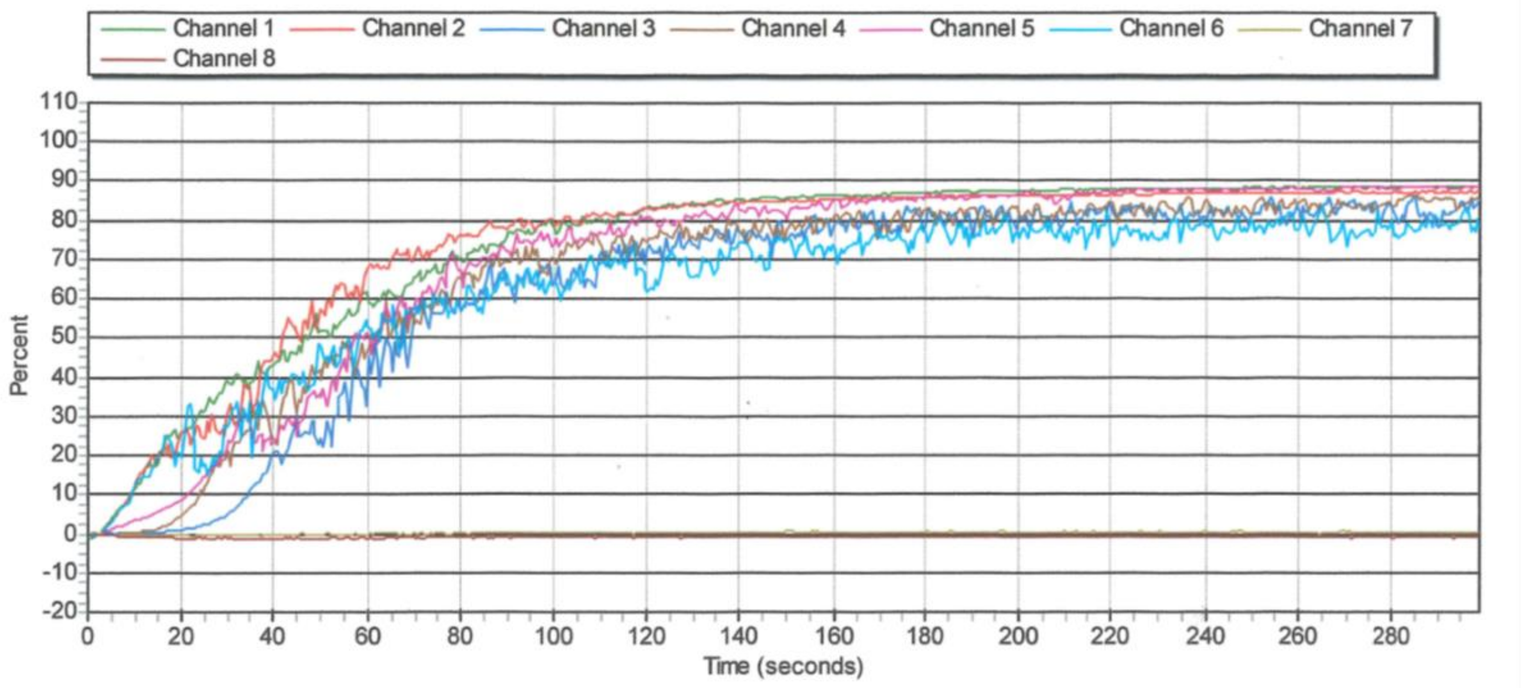

Table S1. Summary of patient test findings. LTA results were obtained from historical records, with information on ages, sex. Diagnoses were obtained by review of electronic medical records. Ages at first test are shown. Abbreviations for diagnoses listed below the data for samples with platelet count below ≤80 X 10^9 platelets/L. Abbreviations in headers: years, yrs; CBC plt, complete blood count platelet count; PRP Plt, platelet rich plasma platelet count; 0 (U); non-diagnostic false positive with U46619; NSAID?, suspected NSAID-induced abnormalities. Within test outliers are indicated (red highlighted cells).

Patient samples with 240-249 X 10^9 platelets/L

							Information on whether agonist responses were normal (0) or abnormal (1) for the sample platelet count and the % maximal aggregation (MA)																			
							1.25 mg/mL ristocetin		0.5 mg/mL ristocetin		5 µg/ml Collagen		1.25 µg/ml Collagen		5 µM ADP		2.5 µM ADP		1.6 mM arachidonic acid		1 µM thromboxane analog U46619		6 µM epinephrine			
Code	sex	Age yrs	# tests	CBC Plt x10^9/L	MPV	PRP Plt x10^9/L	Result	MA	Result	MA	Result	MA	Result	MA	Result	MA	Result	MA	Result	MA	Result	MA	Result	MA	LTA Findings 0=Normal 1=abnormal	Diagnosis
P3079	M	12	1	128	7.5	240	1	75	1	2	1	79	1	78	1	77	1	38	1	82					0	TCP, unknown
P3079				128		240					1	82														cause
P3079				128		240					1	81														
P3070	F	16	1	202	10.2	240	1	90	1	4	1	93	1	68	1	64	1	55	1	98	1	86	1	86	0	Non-diagnostic
P3070						240									1	71										investigations
P3070						240																				
P21417	M	17	1	101	10.7	241	1	83	1	2	1	84			1	83	1	82	1	83	1	82	1	84	0	Suspected ITP
P21417						241					1	83														
P30015	F	30	1	172	8.6	243	1	74	1	3	1	84	1	84	1	84	1	76	1	85	1	83	1	88	0	Non-diagnostic
P30015				172		243		74			1	85														investigations
P30015				172		243					1	86														
P50317	F	35	1	127	9.4	246	1	87	2	93	1	90	1	93	1	94	1	95	1	95	1	95	1	91	1	Suspected PT VWD
P50317						246				92																
P50317						246				87																
P3019	F	42	1	139	9.9	247	1	97	1	1	1	98	1	96	1	91	1	53	1	97	1	88	1	94	0	NA
P3019						247															1	95	1	91		

Patient samples with 141-239 X 10^9 platelets/L

Information on whether agonist responses were normal (0) or abnormal (1) for the sample platelet count and the % maximal aggregation (MA)																								
							1.25 mg/mL ristocetin		0.5 mg/mL ristocetin		5 µg/ml Collagen		1.25 µg/ml Collagen		5 µM ADP		2.5 µM ADP		1.6 mM arachidonic acid		1 µM thromboxane analog U46619			
Code	sex	Age yrs	# tests	CBC Plt x10^9/L	MPV	PRP Plt x10^9/L	Result	MA	Result	MA	Result	MA	Result	MA	Result	MA	Result	MA	Result	MA	Result	MA	LTA Findings 0=Normal 1=abnormal 2=abnormal with one agonist, uncertain significance	Diagnosis
P3042	F	28	1	92	8.5	142	1	87	1	4	1	88	1	16	1	48	1	27	1	86	1	60	0	Non-diagnostic
P3042						142							1	18							1	72		investigations
P3059	M	74	1	65	7.7	132	1	75	1	1	1	85	2	2	1	44	1	29	1	13	1	38	0	NA
P3059						132	1	78			1	85			1	42					1	37		(external)
P50644	F	43	1	78	13.3	142	1	76	1	1	1	76			1	36	1	22	1	78	2	23	0 (U)	ITP (2 other tests
P50644						142									1	36	1	19			2	19		excluded for WBC contamination)
TCP019	F	53	2	129	9.8	143	2	13	1	3	1	72	2	4	1	68	1	53	1	62	1	77	1	Transient acquired
TCP019						143	2	15			1	72	2	7					1	63				BSS
TCP019				204		241	1	92	1	2	1	88	1	54	1	89	1	88	1	86	1	87	0	
P30957	F	26	1	81	10.3	144	1	74	1	1	1	74			1	30	1	16	1	72	2	37	0 (U)	ITP
P30957						144	1	66			1	74			1	33	1	20	1	63				
P41179	M	80	1	174	8.6	145	1	77	1	1	1 outlier	68	1	60	1	60	1	40	1	73	1	75	0	MDS
P41179						145					1	82												
P41179						145					1	81												
P3058	M	66	1	69	9.3	145	1	68	1	1	1	64	1	54	1	39	1	28	1	60	2	19	0 (U)	NA (external)
P3058						145	1	65			1	69			1	45					2	12		
P3058						145															1 outlier	66		
P3072	M	52	1	133	7.8	146	1	75	1	4	2	3	2	3	1	87	1	80	2	1	2	15	1	MDS, 2° PFD
P3072						146	1	84			2	7	2	7					2	6	2	20		with DGD
P3072						146	2 outlier	30																
P50945	M	11	2	88	8.4	146	1	81	1	2	1	86	1	59	1	62	1	68	1	77	2	9	0 (U)	TCP, unknown cause
P50945				71	9	127	1	76	1	7	1	77			1	31	1	17						
P3033	F	74	1	79	7.9	147	1	84	1	2	1	60	2	4	1	52	1	41	2	8	1	78	1	TCP, unknown
P3033						147													2	8	2 outlier	18	(NSAID)	cause
TCP107	F	55	2	75	8.9	150	2	50	1	0	2	45	2	24	2	24	1	15	1	51	2	7	1	PFD, unknown
TCP107						150	2	56			2	45											1	cause with TCP
TCP107				75	8.7	153	1	66	1	2	1	74	1	32	2	32	1	22	1	70	2	0		
TCP107						153	1	69	1	3	1	76	1	38	1	39	1	24		70	2	3		
P3061	F	24	1	127	13.6	151	2	36	1	0	2	44			2	25	1	22	1	37	2	34	1	catastrophic APS
P3061						151	2	43			2	43			2	27			1	41	2	35		
P3050	F	13	1	86	9	153	1	92	1	5	1	91			1	45	1	23	1	87	2	25	0 (U)	ITP
P3050						153											1	29			2	21		
P3040						153											1	23			2	26		

P3044	M	85	1	95	9	153	1	80	1	5	2	58	2	14	1	84	1	76	18	9	1	53	1	PFD, unknown
P3044						153					2	61	2	11					27		1	61		cause with DGD
P3077	M	17	1	98	9.3	153	1	86	1	1	1	94			1	67	1	22	1	89	1	81	0	TCP, unknown
P3077						153									1	47	1	21						cause
P3077						153									2- outlier	36								
TCP012	M	66	2	81	7.2	155	2	14	1	3	2	8			2	1	2	0	2	14	2	3	1	CLL, acquired PFD,
TCP012				65	7.7	130	2	36	1	5	2	11			2	6	2	0	2	24	2	5		Other samples
TCP012						130					2	5												
P50306	F	57	1	77	10.5	155	1	72	1	1	1	74			1	70	1	61	1	75	1	48	0	TCP, unknown
P50306						155					1	77									1	42		cause
TCP068	N	47	1	106	7	155	1	67	1	0	2	3			1	54	1	34	2	0			1	PFD (familial)
TCP068						155					2	4							2	0				With DGD
P30086	F	76	1			155	1	76	2	84	1	91	1	91	1	91	1	89	1	78	1	84		Suspected
P30086						155			2	73									1	76				PT VWD
P30086						155			2	64														
P3040	F	42	1	129	9.7	156	1	81	1	4	1	90	1	77	1	53	1	37	1	89	1	84	0	NA (external)
P3040						156							1	72										
TCP005	F	64	1	110	7.9	156	1	66	1	1	1	64	1	61	1	61	1	60	1	59	1	63	0	TCP from cirrhosis
TCP005						156	1	64			1	64	1	62					1	59	1	64		
P30629	F	27	2	13	11.1	23		54	1	1	1	45	1	48	1	38	1	22	1	40	1	42	0	TCP, unknown
P30629						23			1	0														cause (familial)
P30629						23			1	0														
P30629				84	12.9	158	1	77	1	0	1	87	1	89		90	1	89		84		81	0	
P30629						158																		
P30629						158																		
P20913	M	84	1	106	9.6	158	2	67	1	2	1	75	1	76	1	65	1	74	1	75	1	75	1	TCP from
P20913						158	2	65																cirrhosis
P10705	F	45	2	115	12.7	162	1	73	1	9	1	82			1	38	1	22	1	82		57	0	TCP, unknown
P10705						162			1	9					1	37						51		cause (familial)
P10705						162			1	11														
P10705						162			2 outlier	14														
P10705						156	1	74	1	5		78			1	23		15	1	76		41	0	
P10705						156										37		18				52		
TCP037	F	33	2	112	9.5	164	1	95	1	1	1	92			1	75		75	1	91	1	92	0	TCP. unknown cause (familial)
TCP037				106		161	1	96	1	1	1	98			1	78	1	60						
p3053	F	41	2	85	10.0	163	1	73	1	0	2	26			1	54	1	34	1	80	2	2	1	Acquired
p3053						163					2	29									2	2		thrombocytopenia
p3053				81	10.0	157	1	84	1	3	1	86			1	64	1	48			2	16		
p3053						157															2	27	0 (U)	
p3053						157															2	17		
P3054	F	25	1	97	10.7	164	1	71	1	1	1	82			1	38	1	23	2	4	1	67	1	NA
P3054						164													2	9			NSAID?	
P21418	F	54	1	90	8.3	165	1	79	1	2	1	84			1	80	1	83	1	84	1	84	0	ITP
P21418						165					1	84												
P21418						165					1	84												
P40277	F	0.5	2	125	12.3	165	1	81	1	0	1	91			1	90	1	87			1	86	0	Jacobsen syndrome
P40277				124	13	222	1	78	1	1	1	89		87	1	84	1	81	1	88	1	87	0	

P30631	F	33	1	97	10	167	2	62	1	1		90		87		87		88		88		86	0	ITP
P30631						167	1	71																
TCP055	F	53	1	93	9.8	168	1	100	1	1	1	98			1	52	1	36	1	97	1	93	0	ITP
TCP055						168									1	52	1	34						
P30406	F	41	1	126	12	169	1	79	1	1	1	90	1	87	1	89	1	82	1	90	1	89	0	ITP
P30406						169	1	82																
P30406						169	1	77																
TCP033	F	65	1	121	7.5	170	1	91	1	4	1	89			1	58	1	38	1	86	1	68	0	NA
TCP033						170															1	75		
P10320	M	12	2	94	7.5	174	1	88	1	0	1	90	1	75	1	61	1	50	2	16	1	80	2	TCP, unknown cause
P10320						174													2	20			NSAID?	(familial)
P10320						190	1	87	1	0	1	86	1	60	1	66	1	42	1	89	2	16		
P10320						190															2	21	0 (U)	Additional tests at
P10320						190															2	20		lower counts
P3045	M	12	1	171	7	172	2	5	1	2	1	80	1	77	1	79	1	74	1	73	1	75	0	
P3045						172	2	5			1	82							1	78				Non-diagnostic
P3045						172	2	6											1	82				investigations
P3045						172	1 outlier	72											1	80				
P50800	F	30	1	123	8.2	174	1	85	1	4	1	88			1	87	1	90	1	88	1	90	0	Gestational TCP
P50800						174															1	90		
p3022	F	57	2	100	10.4	175	1	83	1	9	1	78			1	56	1	37	1	79	1	78	0	NA
p3022						175	2 outlier	67									1	39						
p3022				104	10.6	165	1	87	1	5	1	90			1	63	1	36	1	89	1	73		
p3022						165															1	76		
TCP102	F	31	2	87	12.8	177	2	39	1	1	2	44			2	20	2	10	2	42	2	12	1	TCP, unknown
TCP102						177	2	38																cause (familial)
TCP102				78	13.4	116	1	79	1	1	1	84			2	46	1	20	1	84	1	82	2	
TCP102						116					1	85				44	1	24						
P50394	F	51	2	110	11.2	180	1	92	1	1	1	93	1	90	1	92	1	91	1	94	2	13	0	TCP, unknown
P50394						180															2	9		cause (familial)
P50394		43		85	9.2	143	1	92	1	0	1	94			1	87	1	60	1	92	1	87	0	
p3029	F	25	1	112	10.8	180	2	42	1	2	1	73	1	74	1	70	1	72	1	71	1	76	1	2M VWD (delayed RIPA
p3029						180	2	26			1	76	1	71					1	73	1	74		affected MA)
p3029						180	2	14																
TCP008	M	54	1			181	1	92	1	6													0	Known VWD
TCP008						181	1	86	2	11														
P726	M	74	1	115	7.6	182	1	70	1	0	1	79	1	76	1	60	1	32	1	73	1	73	0	TCP from
P726						182	1	78			1	81							1	71				cirrhosis
P726						182	1	80																
P3049	M	64	1	79	11.2	182	2	27	1	2	2	41	1	42	1	39	1	38	2	41	2	40	1	PFD (familial)
P3049						182	2	36																unknown cause
P3049						182	2	31																
P20071	M	77	1			183	1	86	1	1	1	93	2	32	2	38	1	14	1	92	1	68	0	MDS
P20071						183									2	32	1	16						
P20071						183									2	31								
P50917	F	52	1	103	9	184	1	83	1	1	1	89	1	86	1	88	1	89	1	88	1	88	0	ITP
P50917						184	1	80							1	90	1	90						
p3066	F	53	1	100	8.9	185	1	86	1	2	1	82	2	39	1	67	1	43	1	84	1	83	2	Bleeding problems,
p3066						185					1	81	2	24										unknown cause
p3066						185					1	85												
TCP032	M	40	1	150	7.6	186	1	94	1	2	1	92			1	43	1	22	1	87	1	86	0	TCP,
TCP032						186									1	41	1	23						disproportionate bleeding

P40596	F	60	2	128	7	187	1	59	1	1	2	57			1	46	1	37	1	61	2	14	1	FPDMM
P40596						187					2	58									2	20		
P40596				126	7.1	217	1	72	1	0	2	69			1	71	1	58	1	80	2	4		
P40596						217	1	80			2	68									2	6		
P41323	M	75	2	103		184		58			2	46							2	5	2	16	1	Lymphoma, 2°
P41323				88		170		65		3	2	56			1	32	1	19	1	67	2	24		PFD with DGD
P41323						170		73			2	61			1	33					2	32		
TCP071	M	29	1	88	10	186	1	84	1	2	1	92	1	90	1	48	1	36	1	88	1	95	2	ITP
TCP071						186									1	40	1	27						
TCP071						186									2 outlier	35	1	25						
P50925	F	24	1	144	11.8	187	1	84	1	1	1	90	1	81	1	81	1	44	1	93	2	8	0 (U)	ITP
P50925						187															2	8		
P50925						187															2	10		
TCP108	M	33	2	71	9	135	1	81	1	6	1	81			2	19							1	TCP, unknown
TCP108						135									2	18								
TCP108				112	8.8	188	1	83	1	7	1	76	2	4	2	25	2	9	1	56	2	7	1	cause
TCP108						188					1	80	2	12	2	29	2	7			2	0		
P20605	F	21	1	90	11.2	188	2	57	1	2	1	61			1	60	1	51		61	1	60	2	TCP, unknown
P20605						188	2	56																cause (familial); false positive RIPA?
TCP011	F	14	1	103	11.1	188	1	87	1	2	1	89	1	93	1	85	1	45	1	92	1	87	0	ITP
TCP011						188	1	87	1	1														
TCP011						188	1	87																
P3025	M	36	2	163	8.4	189	2	56	1	3	1	63	2	2	2	24	2	11	2	4	2	11	1	PFD, unknown
P3025						189	2	66			1	65	2	4	2	22	2	16	2	11	2	16		cause
P3025						153	2	21	1	2	2	22	2	13	2	13	2	8	2	21	2	20		
P51619	M	15	1	141	8.8	189	1	81	1	1	1	90			1	85	1	29	1	87	2	4	0 (U)	ITP
P51619						189															2	15		
P10989	F	31	1	101	9.2	190	1	75	1	2	1	77			1	65	1	40	1	77	2	16	0 (U)	TCP from liver
P10989						190															2	4		disease
P3063	F	70	1	86	10.5	190	1	90	1	4	1	91	1	60	1	85	1	50	1	89	1	88	0	TCP, unknown
P3063						190							1	71										cause
TCP077	M	90	1	110	8.7	191	1	90	1	2	1	80			2	25	1	28	1	70	1	71	2	TCP from suspected
TCP077						191					1	81			2	26			1	64				MDS
TCP098	F	71	1	94	9.4	193	1	82	1	1	1	95	1	94	1	94	1	92	1	95	1	95	0	ITP
TCP098						193	1	90																
P51040	M	64	1	127	7.5	193	1	73	1	1	1	76	2	19	1	51	1	31	1	76	2	49	0	Non-diagnostic
P51040						193	1	62			1	75												Investigations
P51040						193					1	72												
P3006	F	63	1	114	11.1	193	1	73	1	7	1	87	1	73	1	79	1	66	1	86	1	80	0	TCP from cirrhosis
P3006						193	1	87					1	66										
P3006						193	1	85					2 outlier	53										
P40970	M	66	1	96	11.4	196	1	85	1	3	1	90			1	89	1	88	1	89	1	88	0	TCP from cirrhosis
P40970						196			1	11														
P3024	F	33	1	127	11.7	196	1	80	1	1	1	93			1	94	1	90	1	95	1	97	0	NA
P3024						196	1	85																
TCP066	F	85	1	116	8.6	197	1	85	1	0	1	80			1	79	1	75					0	TCP from liver disease
TCP066						197					1	81												
P3062	F	45	1	112	9.3	198	1	78	1	1	1	90			1	63	1	39	1	83	1	85	0	Non-diagnostic
P3062						198													1	85				investigations
P3062						198													1	82				
P3039	M	15	1	91	10.7	199	1	81	1	1	1	90	1	90	1	89	1	89	1	87	1	88	0	TCP, unknown
P3039						199									1	93								cause (familial)

P10331	F	77	1	107	10.3	199	1	75	1	1	1	85	1	85	1	84	1	82	1	86	1	85	0	ITP
P10331						199	1	68		4														
TCP054	M	17	2	122	10.4	160	1	72	1	3	2	3			2	0	2	0	2	1	2	0	1	PFD, unknown
TCP054						160					2	1			2	14	2	0						cause with TCP
TCP054				125	9.5	200	1	76	1	3	1	84	2	3	2	21	2	9					2	
P3055	M	20	1	116	7.4	200	1	82	1	3	1	82			1	65			1	60	2	19	0 (U)	TCP, unknown
P3055						200					1	81									2	15		cause
P31180	M	65	1	105	10.9	202	1	74	1	3	1	83			1	78	1	52	1	80	1	82	0	TCP, drug-
P31180						202															1	84		induced
P31180						202															2 outlier	16		
p3016	F	91	1	131	9.8	201	1	72	1	3	1	76	1	70	1	73	1	53	1	79	1	76	0	NA
p3016						201	1	73																
TCP090	F	49	1	141	8.9	202	1	89	2	88	1	99			1	73	1	56	1	92	1	90	1	Type 2B VWD
TCP090						202			2	88														
TCP087	F	36	2	142	7.5	204	1	81	1	2	1	80			1	44	1	46	1	74	1	61	0	Other TCP
TCP087						204	1	70			1	72			1	48			1	72	1	59		plus Cushing's from
TCP087						150	1	85	1	4	1	74			1	42	1	33	1	54	1	51		a tumor
TCP087						150					1	74					1	35	1	56				
P40169	F	44	1	107	10	208	1	81	1	10	1	85			1	79	1	75	1	83	1	84	0	TCP from liver
P40169						208				13														disease
P40169						208				11														
P40169						208			2 outlier	48														
P3007	F	23	1	109	9.7	208	1	76	1	1	1	88	1	61	1	59	1	35	1	84	2	24	0 (U)	TCP, unknown
P3007						208							1	62							2	21		cause (familial)
P3007						208							1	67										
P3013	F	48	2	158	8	208	1	90	1	3	1	84			1	74	1	62	1	82	1	82	0	Bleeding
P3013						208					1	84												problems
P3013						227					1	70												of unknown cause
P3013						227					1	71												
TNA30	F	29	1	122	12.2	210	1	66	1	3	1	71		14	1	66	1	54	2	10	1	72	1	NSAID-induced
TNA30						210	1	64			1	74		20					2	20				platelet dysfunction
P20497	M	10	1	126	11	210	1	84	1	2	1	89	1	58	1	83	1	89	1	85	1	65	0	ITP
P20497						210															1	66		
P20497						210															1	71		
P3073	F	7	1	192	7.9	211	1	85	1	2	2	40	2	1	1	56	1	38	2	2	1	91	1	Post-radiation
P3073						211					2	40	2	2					2	2				vasculopathy on ASA
TCP027	F	30	1	103	8.6	211	2	60	1	2	2	63			1	45	1	23	1	62	1	61	1	TCP, unknown
TCP027						211	2	61																cause (familial)
TCP040	M	14	1	146	9.9	215	1	74	1	1	1	81			2	24	2	16	1	79	2	23	1	ITP
TCP040						215													1	76	2	20		
TCP040						215															2	21		
P11569	F	81	2	149	8.9	215	1	75	1	0	1	80			1	63	1	25	2	1	2	12	1	PFD, RUNX1 VUS
P11569						215													2	3	2	8		
P11569						217	1	76	1	1	1	80			1	70	1	46	2	9	2	4	1	
P11569						217													2	1	2	0		
P30539	F	25	1	159	10.8	216	1	86	1	1	1	91	1	91	1	8	1	56	1	92	1	92	0	Non-diagnostic
P30539						216	1	83																Investigations
TCP057	F	46	2	124	10.5	162	1	86	1	2	1	89			1	81	1	72	1	87	1	89	0	TCP, unknown
TCP057				156		218	1	87	1	5	1	88	1	92	1	90	1	68	1	86	1	91		cause with disproportionate bleeding
P3028	F	17	1	168	9.8	220	1	91	1	1	1	91	1	80	1	44	1	33	1	82	1	75	0	NA (EXTERNAL)
P3028						220									1	48								
P51203	M	40	1	129	6	222	1	70	1	3	1	71	2	40	1	59	1	43	1	66	2	28	1	FPDMM
P51203						222	1	68			1	74	2	44					1	69	2	34		
P3041	F	10	1	125	7.3	227	1	75	1	2	2	50	2	3	1	73	1	60	1	82	2	0	1	FPDMM
P3041						227	1	76													2	1		

P10874	M	9	1	129	8.9	223	1	79	1	0		84	1	81	1	55	1	42	1	84	1	80	0	Non-diagnostic
P10874						223									1	73								Investigations
P10874						223									1	62								
P20126	F	67	1	110	11.1	224	1	77	1	1	1	61			1	71	1	64	1	77	1	61	0	Non-diagnostic
P20126						224					1	71												investigations
P3034	M	17	1	90	NA	224	1	74	1	1	1	80			1	80	1	79	1	76	1	76	0	TCP unknown
P3034						224	1	78																cause (familial)
P3034						224	2 outlier	0																
P3064	N	74	1	160	8.6	230	1	83	1	2	1	76	2	3	1	56	1	33	1	76	1	76	2	MDS
P3064						230					1	78	2	17										
p386	F	28	2	141	6.9	230	1	88	2	14	1	88			1	87	1	70	1	83	2	28	1	TCP, unknown
p386						230			2	14											2	35		cause (familial)
p386				118	6.9	224	1	76	2	14	1	71			1	78	1	51	1	65	2	26		
p386						224			2	12											2	26		
P3071	M	61	1	279	5.8	227	1	101	1	0	2	20			1	108	1	104	1	86	1	96	2	PFD 2° to MDS
P3071						227					2	25												acquired DGD
P3071						227					2 outlier	11												
P40922	M	45	1	116	9	229	2	68	1	2	1	91	1	75	1	74	1	35	1	92	1	86	0	QPD
P40922						229								80										False positive with
P40922						229							2 outlier	29										ristocetin
P21753	F	12		133	9.6	180	1	74	1	3	1	71	1	57	1	25	1	16	1	66	1	59	0	TCP, unknown
P21753			2	152	9.7	230	1	72	1	1	1	82	1	74	1	52	1	22	1	84	1	72	0	cause (familial)
P21753						230	1	79			1	83			1	61	1	21			2	13	0	additional lower
P21753						230		73				76			2 outlier	39								platelet count
P21753				121	11.3	99	1	73	1	11	1	76	1	26	0								0	samples
P1753R						99	1	76	1	11	1	79	1	37										
P10801	M	40	1	132	7.2	232	1	80	1	1	1	91	1	80	1	53	1	33	1	92	2	15	0 (U)	NA (external)
P10801						232									1	57	1	37			2	25		
P3017	F	32	1	181	8.1	233					1	71		45									0	NA
P3017						233					1	77												
P3032	F	62	1	118	13.1	233	1	81	1	1	1	90			1	80	1	63	1	88	2	37	0	Non-diagnostic
P3032						233															2	57		investigations
P3032						233															2	47		
P811	M	64	1	118	7.6	236	1	87	1	0	1	92	1	83	1	76	1	41	2	8	2	19	1	Familial PFD unknown cause (normal count
P811						236	1	81			1	87							2	10	2	1	1	PRP abnormal with
P811						236													2	12				multiple agonists)
P21092	F	7	1	182	11.9	237	1	83	1	2	1	89	1	84	1	86	1	72	1	90	1	89	0	non diagnostic
P21092						237			1	8														investigations
P21092						237			1	1														
P31419	M	38	1	140	10.6	238	1	79	1	2	1	86			1	79	1	75	1	81	1	80	0	TCP, unknown cause
P31419						238	1	73											`					HHT
P31419						238	2 outlier	69																
TCP039	M	55	1	132	7.9	239	1	84	1	2	1	79	1	78	1	86	1	69	2	33	1	90	1	TCP unknown cause
TCP039						239							2	45					2	31				plus NSAID

Patient samples with 81-140 X 10^9 platelets/L

							Information on whether agonist responses were normal (0) or abnormal (1) for the sample platelet count and the % maximal aggregation (MA)									
							1.25 mg/mL ristocetin		0.5 mg/mL ristocetin		5 µg/ml collagen		5 µM ADP			
Code	sex	Age yrs	# tests	CBC Plt x10^9/L	MPV	PRP Plt x10^9/L	Result	MA	Result	MA	Result	MA	Result	MA	LTA Findings 0=Normal 1=abnormal 2=abnormal with one agonist, uncertain significance	Diagnosis
A139	F	29	2	63	8.6	94	2	52	1	1	2	54	2	24	1	Familial PFD with TCP
A139				35	8.1	70	2	42	1	2	1	63	2	12		
P3003	M	38	2	45	10	82	1	74	1	4	2	41	1	61	0	MDS, acquired PFD with
P3003						82					2	33				DGD, pathogenic
P3003				40	9.9	82	1	61	1	3	2	19	1	51	1	RUNX1
P3003						82	1	64			2	10				mutation
P3065	F	53	1	52	8.1	83	1	66	2	25	1	56	1	45	1	NA (external)
P3065						93			2	28						
P3043	F	22	1	58	12.7	83	1	87	1	8	1	94	1	39	0	ITP
P3043						83							1	48		
P3076	F	71	1	78	NA	83	2	10	1	11	1	89	1	86	1	BSS
P3076						83	2	10	1	15						
P3076						83	2	11	1	16						
TCP095	F	65	1	53	8.5	83	1	79	1	4	1	73	2	18	2	ITP
TCP095						83							2	13		
TCP063	F	6	1	57	7.6	85	2	56	1	2	2	57	2	18	1	ITP
TCP063						85	2	54			2	46	2	23		

P3023	F	55	1	61	9.7	85	2	43	1	5	1	58	2	47	0	TCP, unknown cause
P3023						85	2	41					2	51		
P3023						85	2	35								
P11622	F	56	1	46	NA	86	2	1	1	0	2	17	2	5	1	ITP with acquired BSS and
P11622						86	2	1	1	0	2	17	2	4		acquired GT
P41548	F	63	1	45	10.7	87	2	58	1	3	1	59	1	54	0	TCP from liver disease
P41548						87	1	61			1	60				
P3036	F	25	2	97	13.9	133	2	30	1	3	1	86			2	NA (external)
p3036						133	2	25							0	
p3036						133	2	11								
p3036				87	11.5	90	1	93	1	4	1	91	1	81	0	
p3036						90	1	92	1	4						
P40061	F	48	1	58	NA	91	2	55	1	0	2	61	2	9	1	TUBB1-RT
P40061						91	2	52			2	58	2	10		
P11567	F	35	1			91	1	80	1	2	1	80	1	85	0	TCP, unknown cause
P11567						91							1	74		(suspected: familial)
P3056	M	54	1	64	8.5	92	2	3	1	1	2	45	2	13	1	ITP with inhibitory
P3056						92	2	2					2	14		antibodies
P50283	M	53	1	64	8.2	92	2	51	1	0	1	60	1	46	1	TCP from liver disease
P50283						92	2	48			1	62				
P50283						92	2	49			1	64				
P3030	F	85	1	77	7.4	93	2	54	1	1	2	13	1	21	1	TCP, acute blood loss
P3030						93	2	55			2	13	1	21		
P20106	F	36	2	147		96	1	62	1	5	1	63	2	12	2	ITP with platelet
P20106						96	1	69	1	4	1	60	2	9	2	glycoprotein antibodies
P20106				71	10.2	53	2	17	1	11	2	8			1	
P20106						53	2	20	1	7	2	7				
P20106						53	2	25	1	12	2	7				
P3067	M	31	1	45	9.5	98	1	82	1	4	1	81	1	66	0	TCP, unknown cause
P3067						98	1	70								
P3067						98	1	63								

P21753	F	18	2	121	11.3	99	1	73	1	11	1	76	1	26	0	TCP, unknown cause (familial)
P21753						99	1	76	1	11	1	79	1	37		Additional samples, other counts
P30936	M	5	1	60	11.9	102	2	0	1	0	1	87	1	85	1	BSS
P30936						102	2	0	1	2						
P30936						102	2	1								
P30936						102	2	0								
p3047	M	72	1	61	NA	105	1	60	1	1	2	44	1	40	2	TCP, unknown cause
p3047						105					2	46				
P21632	F	46	1	53	13.4	105	1	62	2	65					1	Type 2B VWD
P21632						105			2	66						
P3037	M	71	1	76	9.2	105	1	68	1	1	2	33	1	57	2	NA
P3037						105	1	67			2	40				
P3037						105	2 outlier	58			2 outlier	6				
P50464	F	41	1	66	12.2	107	1	73	1	1	2	57	2	9	1	ITP
P50464						107					2	54	2	9		
P3068	M	55	1	68	12	108	2	41	1	2	1	85	1	83	1	TCP, unknown cause
P3068						108	2	45	1	2						(familial)
P03095	M	74	1	84	7.2	109		21	2	38	1	64	1	59	1	MDS, paradoxical ristocetin
P03095						109		15	2	49						findings
P03095						109		16	2	50						
P3005	M	34	1	56	na	110	1	73	1	4	1	73	1	57	0	Gray platelet syndrome
P3005						110		71								
P11558	F	18	1	60	10.5	111	1	72	1	4	1	86	1	85	0	TCP, unknown cause
P11558						111	1	72								(familial)
P11558						111	1	73								
P3010	F	43	2	94	9	111	2	51	1	4	2	52	2	13	1	TCP from liver disease
P3010						111	2	55	1	5	2	46	2	16		
P3010						104	2	57	1	2	2	35	2	16	1	
P3046	F	32	1	80	NA	112	2	54	1	1	1	63	1	61	2	TCP, unknown cause
P3046						112	2	55			1	64				(familial)
P3046						112	2	54			1	65				

P3031	F	32	1	75	9.9	112	1	72	1	4	1	86	1	32	0	ITP
P3031						112		83						31		
P21608	F	46	2	63	10.9	116	2	40	1	2	1	80	1	66	2	ITP
P21608						116	2	37					1	60		
P21608						116	2	22					1	92		
P21608				81	14	127	2	42	1	7	1	90			2	
P21608						127	2	37								
P11366	F	15	1	70	12.8	117	1	70	1	1	1	81	1	35	0	ITP
P11366						117	1	67			1	80	1	36		
TCP114	F	35	1	62	11	118	1	84	1	5	1	82	1	74	0	ITP
TCP114						118	1	87		6						
P51356	F	69	1	67	7.6	118	1	65	1	0	1	73	1	64	0	ITP
P51356						118	1	71			1	74				
P51356						118	1	60			1	72				
P3009	M	22	1	81	NA	119	1	89	1	2	1	92	1	91	0	TCP, unknown cause
P3009						119	1	86	1	1	1	90	1	92		(familial)
p3020	F	39	1	92	9.8	119	1	87	1	8	1	80	1	34	0	ITP
p3020						119			1	10	1	81	1	27		
P3015	F	60	1	63	9.6	120	1	65	1	2	1	66	1	63	0	MDS
P3015						120	1	60			1	69				
P40097	F	49	2	98	11.4	120	1	87	2	88	1	90	1	98	1	type 2B VWD
P40097						120			2	93						
P40097						121	1	87	2	90	1	90	1	92	1	
P40097						121			2	91						
P31491	M	51	1	67	7.1	121	1	74	1	6	1	69	1	27	0	ITP
P31491						121	1	73	1	5	1	68	1	31		
P3075	M	77	1	79	NA	122	1	69	1	1	1	69	1	39	0	NA
P3075						122	1	67								
TCP053	F	25	1	91		122	1	100	1	6	1	67	1	52	0	TCP, unknown cause
TCP053						122					1	73				(familial)
P11748	F	45	1	93	6.4	123	1	79	1	2	1	83	1	79	0	TCP, unknown cause
P11748						123					1	84				
P11748						123					1	85				

P3057	F	13	1	66	NA	128	1	60	1	1	1	67	1	62	0	ITP
P3057						128	1	62			1	67				
P3057						128	1	62			1	67				
TCP092	F	29	1	72	10.4	128	1	85	2	33	1	87	1	71	1	Type 2B VWD
TCP092						128			2	33						
P51088	M	16	1	76	6.6	129	1	84	1	2	2	1	2	17	1	ITP
P51088						129					2	2	2	21		
TCP103	F	55	1	100	7.2	130	2	72	1	3	2	51	1	27	0	TCP from liver disease
TCP103						130	1	54	1	2	1	65	1	30		
P30869	F	56	1	76	11.8	130	2	30	1	0	2	39	1	33	1	ITP
P30869						130	2	31			2	36	1	34		
TCP012	M	66	1	65	7.7	130	2	36	1	5	2	11			1	Acquired PFD, CLL
TCP012						130					2	5				Additional tests, other counts
P3078	M	76	1	89	8.7	131	1	71	1	5	2	31	1	62	2	MDS
P3078						131	1	63			2	32				
TCP036	F	45	1	71	9.9	134	1	85	1	2	1	85	1	36	0	ITP
TCP036						134							1	43		
TCP036						134							1	43		
P3038	F	53	3	82	8.3	135	2	45	1	4	2	44	1	41	1	APS
P3038						135	2	47								
P3038				81		130	2	52	1	4	2	52	2	19		
P3038						100	1	93	1	2	1	86	1	46	0	
P3014	F	11	1	86	7.1	138	2	38	1	2	2	35	2	10	1	Unknown
P3014						138	2	38	1	2	2	35				
P3051	F	32	3	72	6.1	138	1	83	1	2	2	55	1	56	2	TCP, unknown cause with
P3051						119	1	75			2	57	1	40		disproportionate bleeding
P3051						116	1	61	1	6	2	52	1	41		
P3051						116	1	65			2 outlier	28	1	34		
P3069	F	71	1	90	8.5	139	1	87	1	1	1	77	1	62	0	NA (external)
P3069						139					1	88	1	51		

Samples with ≤80 X 10^9platelets/L

							Information on whether agonist responses were normal (0) or abnormal (1) for the sample platelet count and the % maximal aggregation (MA)							
							1.25 mg/mL ristocetin		0.5 mg/mL ristocetin		5 µg/ml collagen			
Code	Sex	Age, yrs	# tests	CBC Plt x10^9/L	MPV	PRP Plt x10^9/L	Result	MA	Result	MA	Result	MA	LTA Findings 0=Normal 1=abnormal 2=abnormal with one agonist, uncertain significance	Diagnosis
TCP078	F	34	2	37		9	2	14			1	94	1	BSS
TCP078						9	2	17						
TCP078				29	7.3	62	2	1	1	4	2	21	1	
TCO078						62	2	2	1	2	2	22		
TCP088	F	32	2	11	7.1	10	2	1	1	1			1	Acquired BSS 2° to ITP
TCP088						10	2	1	1	1				
TCP088						10	2	1						
TCP088						63	2	7	1	1			1	
TCP088						63	2	7						
P41149	F	36	1	4	9.1	17	2	0	1	1	1	33	1	BSS, hepatitis C
P41149						17	2	1						
P10103	M	47	1	51		19	2	0	1	1	1	30	1	BSS
P10103						19	2	5						
P20623	F	68	1	50-100	NA	19	2	4	1	1	1	87	1	BSS
P20623						19	2	4						
P20623						19	2	0						
P10676	F	59	1	30-50	11.6	28	2	26	1	3	2	33	1	ITP with glycoprotein antibodies
P10676						28	2	25	1	0	2	32		
P31579	M	15	1	37	10.8	29	2	14	1	8	2	17	1	Suspected ITGA2B/ITGB3-RT with
P31579						29	2	27	1	12	2	9		decreased (29%) αIIbβ3 expression
P31579						29	2	30	1	10	2	24		(lower platelet count sample rejected)

P3000	F	65	2	18	9.9	31	2	46	1	8	2	35	2	TCP, unknown cause (familial)
P3000						31	2	47	1	6	2	35		
P3000						31	2	46	1	8	2	33		
P3000				21		41	1	52	1	7	1	55	0	
P3000						41	1	55	1	5	1	55		
P3000						41	1	60	1	3	1	54		
P41086	F	34	1	25	8	34	1	70		22	1	68	1	ITP
P41086						34				26				
P41086						34				21				
P3027	M	38	1	26	NA	36	2	0	1	1	2	22	1	BSS
P3027						36	2	0	1	0	2	23		
P40703	F	3	2	71	7.7	38	1	64	1	12	2	5	2	TCP, unknown cause (familial) with
P40703						38	1	62			2	11		DGD
P40703				71		42	2	35	1	1	2	25	1	
P40703						42		34						
P3052	M	37	1	20	NA	42	2	35	1	2	2	37	1	TCP, unknown cause
P3052						42	2	37	1	2	2	39		
P3001	F	40	1	28	13.5	43	1	56		3	2	42	0	
P3001						43	1	55	1	4	2	47		ITP
P3074	F	5	1	56	NA	44	2	3	1	9	1	80	1	BSS
P3074						44	2	6	1	10				
P40931	F	43	1	19		45	1	62	1	0	1	72	0	Suspected MYH9RD
P40931						45	1	61						
P3004	M	79	2	30		46	2	23	1	0	2	38		TCP unknown cause,
P3004	M	79				46	2	22	1	1	2	20	1	some tests abnormal
P3004	M	79		48	14	70	1	73	1	1	2	32	0	
P50950	F	81	1	32	10.8	47	1	62	2	40	1	71	1	Type 2B VWD
P50950						47	1	72	2	38				
P51450	M	4	1	53	NA	50	2	1	1	6	1	75	1	BSS
P51450						50	2	1	1	6				
P10320	M	12	2	94	7.5	50	1	88	1	0	1	90	0	TCP, unknown cause (familial)
P10320				101	7.1	51	1	73	1	1	1	88		(also tested at higher platelet counts)
TCP067	F	21	2	50	NA	51	2	3	1	6	1	78	1	BSS
TCP067						51	2	2	1	10				
TCP067						51	2	2	1	2	1	71		
P3012	F	14	1	35	NA	52	2	27	1	0	2	34	1	ITP
P3012						52	2	26	1	1	2	30		

P20106	F	36	2	71	10.2	53	2	17	1	11	2	8	1	ITP with platelet glycoprotein antibodies
P20106						53	2	20	1	7	2	7		(also tested at higher platelet counts)
P20106						53	2	25	1	12	2	7		
P21100	F	59	2	71	NA	53	2	13	1	2	2	9	1	Suspected ITGA2B/ITGB3-RT
P21100				86		57	2	32	1	3	2	21		with decreased (29%) αIIbβ3 expression
P21100						57					2	23		
P50704	M	37	1	78	9.5	56	1	65	1	8	2	36	0	TCP, unknown cause (familial)
P50704						56	1	65	1	12	2	41		
P50704						56			2 outllier	26				
P3002	M	12	1	41	10.9	58	1	64	1	12	1	67	0	CML, normal aggregation findings
P3002						58	1	70		10	1	55		
P40643	F	4	1	34	NA	58	1	57	1	2	1	55	0	ITP
P40643						58	1	57			1	57		
P20246	F	26	1	50-100	11.3	59	2	46	1	0	1	57	0	Suspected MYH9RD
P20246						59	2	44	1	0	1	54		
P20850	F	34	1	66	15.6	61	1	68	1	14	1	88	0	ITP (vs. gestational TCP)
P20850						61	1	71	2	10	1	82		
P21479	M	18	1	30-40		65	2	51	1	2	1	72	0	Gray platelet syndrome
P21479						65	1	63	1	1				
P30068	F	32	1	36	10.4	69	2	25	1	0	2	35	1	ITP with platelet autoantibodies
P30068						69	2	26	1	1	2	30		
P21347	F	49	1	39	9.8	70	1	49	1	0	2	43	2	TCP from liver disease
P21347						70	2	38	1	0	2	45		
p3048	M	18	1	47	NA	72	2	1	1	3	1	88	1	BSS
p3048						72	2	2						
P11036	F	28	1	67	13.3	73	2	53	1	7	1	62	2	Gestational TCP
P11036						73	2	56	1	11	1	63		
P11036						73			1	13				
TCP104	F	44	1	44	7.7	78	2	28	1	0	1	77	2	ITP
TCP104						78	2	31	1	17				
P11631	F	13	1	52	14.1	79	2	46	2	49			1	Type 2B VWD
P11631						79			2	46				

Abbreviations for diagnoses: APS, antiphospholipid syndrome; ASA, acetylsalicylic acid; BSS, Bernard Soulier syndrome; CLL, chronic lymphocytic leukemia; CML, chronic myeloid leukemia; DGD, dense granule deficiency; FPDMM, familial platelet disorder with predisposition to myeloid malignancies; GT, Glanzmann thrombasthenia; HHT, hereditary hemorrhagic telangiectasia; *ITGA2/ITGB3*-RT, *ITGA2/ITGB3*-related thrombocytopenia; ITP, immune thrombocytopenia; MDS, myelodysplastic syndrome; MYH9RD, MYH9-related disorders; NA, could not be assessed; NSAID, non-steroidal anti-inflammatory drugs that inhibit cyclooxygenase 1; PFD, platelet function disorders; PT-VWD, platelet-type von Willebrand disease; QPD, Quebec platelet disorder; RIPA, ristocetin induced platelet agglutination/aggregation; *TUBB1*-RT, *TUBB1*-related thrombocytopenia; TCP, thrombocytopenia; VUS, variant of uncertain significance

Table S2. Historical data for diluted control platelet rich plasma samples that were used as light transmittance platelet aggregometry controls for patient low platelet count platelet rich plasma samples. Abbreviations as defined for table S1.

Control samples with 240-249 X 10^9 platelets/L

		Information on whether agonist responses were normal (0) or abnormal (1) for the sample platelet count and the % maximal aggregation (MA)																		
		1.25 mg/mL ristocetin		0.5 mg/mL ristocetin		5 µg/ml Collagen		1.25 µg/ml Collagen		5 µM ADP		2.5 µM ADP		1.6 mM arachidonic acid		1 µM thromboxane analog U46619		6 µM epinephrine		
Control code	adj PRP Plt x10^9/L	Result	MA	Result	MA	Result	MA	Result	MA	Result	MA	Result	MA	Result	MA	Result	MA	Result	MA	LTA findings 0=Normal/non-diagnostic 1=Abnormal (specify)
C136	240	196		1	1	1	96	1	95	1	97	1	96	1	95	1	95			0
C3	240	1	85	1	1	1	91	1	66	1	90	1	91	1	93	1	92			0
C12	240	1	84	1	1	1	94	1	94	1	92	1	92	1	96	1	91	1	94	0
C14	240	1	96	1	3	1	91			1	81	1	28	1	94			1	20	0
C1	240	1	95	1	2	1	93			1	95	1	91	1	94	1	92	1	96	0
C15	240	1	96	1	2	1	96			1	86	1	50	1	88	1	88	1	55	0
C2	241	1	101	1	3	1	97	1	96	1	92	1	93	1	96	1	98	1	100	0
C1	241	1	86	1	3	1	89			1	87	1	64	1	89	1	64	1	88	0
C7	241	1	95	1	4	1	95	1	94	1	96	1	101	1	95	1	91	1	96	0
C1	242	1	83	1	1	1	92	1	93	1	92	1	93	1	94	1	92	1	95	0
C2	243	1	91	1	1	1	96	1	95	1	90	1	93	1	95	1	95	1	93	0
C2	244	1	85	1	5	1	87			1	87	1	87							0
C13	245	1	91	1	1	1	96			1	90	1	87					1	95	0
C7	246	1	91	1	2	1	96		96	1	95	1	67	1	95	1	94	1	92	0
C6	247	1	78	1	3	1	92		90	1	91	1	87	1	92	1	92	1	89	0
C2	248	1	88	1	3	1	96		83	1	91	1	96	1	94	1	96	1	97	0
C18	249	1	89	1	4	1	93		93	1	93	1	91	1	94	1	93	1	93	0
C3	249	1	85	1	3	1	92		93	1	93	1	91	1	93	1	92	1	93	0

Control samples with 141-239 X 10^9 platelets/L

		Information on whether agonist responses were normal (0) or abnormal (1) for the sample platelet count and the % maximal aggregation (MA)																
		1.25 mg/mL ristocetin		0.5 mg/mL ristocetin		5 µg/ml Collagen		1.25 µg/ml Collagen		5 µM ADP		2.5 µM ADP		1.6 mM arachidonic acid		1 µM thromboxane analog U46619		
Control code	adj PRP Plt x10^9/L	Result	MA	Result	MA	Result	MA	Result	MA	Result	MA	Result	MA	Result	MA	Result	MA	LTA findings 0=Normal/non-diagnostic 1=Abnormal (specify)
C22	141	1	82	1	4	1	88			1	53	1	31	1	78	1	83	0
C2	142	1	85	1	3	1	84			1	36	1	24	1	32	1	84	0
C6	142	1	68	1	4	1	90	1	73	1	66	1	44	1	88	1	83	0
C1	142	1	83	1	3	1	90	1	40	1	47	1	26	1	85	1	66	0
C65	142	1	85	1	1	1	88			1	80	1	56	1	87	1	65	0
C1	143	1	107	1	0	1	112	1	80	1	54	1	31	1	115	1	115	0
C66	143	1	98	1	4	1	90	1	82	1	58	1	29	2	3	1	29	0
C10	144	1	96	1	3	1	91	1	78	1	86	1	58	1	93	1	90	0
C6	144	1	71	1	2	1	89			1	69	1	43	1	86	2	16	0
C67	144	1	82	1	4	1	88	1	74	1	67	1	77	1	88	1	81	0
C78	145	2	54	1	3	1	92			1	89	1	89					0
C79	145	1	94	1	6	1	92	1	79	1	78	1	46	1	74	1	77	0
C77	145	1	88	1	0	1	92	1	88	1	86	1	70	1	92	1	88	0
C94	146	1	90	1	1	1	96	1	90	1	86	1	87	1	94	1	99	0
C16	147	1	90	1	2	1	91	1	84	1	72	1	41	1	66	1	89	0
C16	147													1	62			
C16	147													2 outlier	19			
C32	148	1	79	1	1	1	84	1	87	1	83	1	85	1	86	1	84	0
C19	150	1	94	1	0	1	84	1	30	1	40	1	23	1	86	1	83	0
C7	150	1	92	1	0	1	92			1	91	1	76	1	93	1	87	0
C24	151	1	88	1	3	1	90			1	83	1	70	1	87	1	90	0
C8	153	1	90	1	1	1	94			1	40	1	18	1	89	1	71	0
C16	153	1	96	1	3	1	94			1	92	1	91	1	94	1	95	0
C16	153					1	95											
C23	153	1	92	1	3	1	96	1	91	1	88	1	89	1	94	1	96	0
C2	153	1	91	1	2	1	91	1	83	1	80	1	69	1	91	1	90	0
C6	154	1	83	1	2	1	89	1	61	1	66	1	43	1	82	1	73	0
C60	155	1	94	1	3	1	93			1	85	1	79	1	94			
C46	155	1	85	1	1	1	82			1	86	1	68	1	86	1	86	0
C5	155	1	66	1	3	1	69	1	51	1	65	1	71	1	68	1	65	0
C5	155													1	68			
C6	156	1	83	1	2	1	90	1	83	1	63	1	63	1	88	1	85	0
C6	156	1	75															
C59	156	1	100	1	3	1	92	1	82	1	76	1	38	1	90	1	87	0
C40	156	1	102	1	3	1	96	1	86	1	68	1	26	1	71	1	94	0
C65	156	1	86	1	5	1	89	1	50	1	83	1	85	1	85	1	86	0
C59	156	1	100	1	3	1	92	1	82	1	76	1	38	1	90	1	87	0
C6	158	1	72	1	2	1	94	1	89	1	90	1	89	1	85	1	83	0

C30	158	1	82	1	3	1	84	1	84	1	84	1	82	1	87	1	85	0
C61	159	1	84	1	1	1	89			1	86	1	50	1	83	1	84	0
C19	159	1	92	1	1	1	94			1	90	1	52	1	97	1	90	0
C21	159	1	85	1	3	1	92	1	89	1	89	1	87	1	88	1	90	0
C58	159	1	93	1	5	1	95			1	89	1	86	1	92	1	94	0
C57	159	1	75	1	0	1	88	1	74	1	72	1	36	1	77	1	82	0
C68	160	1	93	1	2	1	88			1	55	1	27	1	87	1	87	0
C25	160	1	96	1	2	1	98			11	43	1	23	1	77	1	64	0
C3	161	1	83	1	4	1	93			1	90	1	95	1	90	1	90	0
C3	161	1	88															
C2	161	1	87	1	4	1	93			1	59	1	45					0
C59	162	1	96	1	5	1	94			1	77	1	37	1	90	1	86	0
C129	162	1	98	1	4	1	98			1	113	1	46					0
C3	162	1	87	1	3	1	87			1	88	1	90	1	87	1	88	0
C59	162	1	96	1	5	1	94			1	77	1	37	1	90	1	86	0
C31	164	1	86	1	3	1	100					1	27	1	102	1	93	0
C1	164	1	94	1	4	1	78			1	83	1	36	1	87	1	84	0
C3	163	1	86	1	1	1	80			1	89	1	85	1	88	1	89	0
C12	165	1	85	1	0	1	95			1	92	1	83	1	96	1	78	0
C16	165	1	91	1	3	1	90			1	94	1	92			1	94	0
C34	165	1	82	1	4	1	90	1	66	1	77	1	43	1	87	1	86	0
C34	166	1	88	1	4	1	88	2	24	1	76	1	30	1	86	1	87	1
C16	165	1	83	1	3	1	92			1	92	1	92			1	94	0
C1	166	1	83	1	3	1	89	2	17	1	86	1	57	1	93	1	89	0
C23	166	1	95	1	3	1	95	1	76	1	91	1	91	1	93	1	96	0
C95	167	1	85	1	2	1	87			1	85	1	52	1	87	1	86	0
C19	167	1	87	1	3	1	91	1	77	1	83	1	58	1	89	1	83	0
C1	168	1	109	1	3	1	105			1	100	1	94	1	148	1	103	0
C2	168	1	84	1	4	1	97			1	69	1	90	1	96	1	96	0
C69	169	1	83	1	3	1	90	1	88	1	83	1	86	1	89	1	92	0
C49	170	1	83	1	4	1	79			1	87	1	88	1	87	1	89	0
C21	170	1	89	1	4	1	89	1	76	1	88	1	67	1	99	1	81	0
C4	170	1	100	1	4	1	94			1	89	1	34	1	99			0
C68	170	1	72	1	1	1	88			1	72	1	57	2	5	1	91	0
C38	170	1	95	1	9	1	95	1	62	1	90	1	43	1	86	1	91	0
C31	173	1	100	1	3	1	94			1	87	1	54					0
C6	174	1	76	1	3	1	94		91	1	86	1	79	1	93	1	87	0
C12	174	1	85	1	0	1	95			1	92	1	83	1	96	1	78	0
C80	174	1	86	1	0	1	94	1	92	1	89	1	59	1	92	1	90	0
C36	174	1	94	1	5	1	94			1	62	1	29	1	85	1	90	0
C5	175	1	97	1	3	1	91	1	83	1	73	1	48	1	87	1	88	0
C3	175	1	88	1	3	1	82	1	89	1	91	1	89	1	91	1	80	0
C81	176	1	85	1	1	1	90			1	86	1	65	1	95	1	86	0
C6	177	1	77	1	3	1	88			1	72	1	84	1	87	1	87	0
C10	177	2	47	1	3	2	54			1	47	1	19	1	50	2	47	0
C5	179	1	90	1	2	1	94			1	57	1	37	1	89	1	83	0
C96	180	1	88	1	4	1	85	1	85	1	62	1	32	1	90	1	90	0
C96	180			1	6			1	84									
C96	180							1	60									
C97	180	1	88	1	0	1	92	1	87	1	90	1	87	1	91	1	90	0
C9	180	1	85	1	3	1	85	1	81	1	68	1	39	1	79	1	82	0
C2	180	1	94	1	2	1	92			1	68	1	43	1	90	1	92	0
C40	180	1	89	1	2	1	94			1	89	1	62					0
C8	180	1	92	1	2	1	99			1	89	1	39	1	87	1	98	0
C35	181	1	76	1	2													0
C3	182	1	90	1	3	1	93	1	94	1	91	1	93	1	95	1	93	0

C30	182	1	82	1	1	1	85	1	88	1	83	1	82	1	86	1	86	0
C77	182	1	86	1	0	1	94	1	92	1	89	1	59	1	92	1	90	0
C138	182	1	88	1	2	1	94	1	94	1	60	1	32	1	95	1	94	0
C40	182	1	97	1	5	1	95			1	77	1	81	1	88	1	89	0
C40	182	1	97	1	5	1	95			1	77	1	81	1	88	1	89	0
C1	183	1	95	1	0	1	86	1	82	1	83	1	84	1	87	1	87	0
C1	183	1	81	1	1	1	91			1	74	1	43	1	93	1	93	0
C41	183	1	86	1	1	1	91			1	74	1	43	1	93	1	93	0
C3	184	1	84			1	92							1	87	1	94	0
C6	184	1	90	1	0	1	93	1	83	1	91	1	82	1	92	1	93	0
C6	184									1	89	1	85					
C23	185	1	104	1	3	1	101	1	98	1	100	1	99	1	100	1	99	0
C4	185	1	81	1	0	1	87	1	86	1	86	1	87	1	80	1	84	0
C99	185	1	93	1	3	1	90			1	81	1	45	1	89	1	87	0
C6	186	1	91	1	3	1	88	1	82	1	69	1	66	1	95	1	88	0
C33	186	1	97	1	4	1	94			1	84	1	51	1	91	1	90	0
C1	186	1	92	1	2	1	93	1	90	1	90	1	93	1	92	1	94	0
C3	186	1	86	1	1	1	92			1	93	1	92	1	92	1	92	0
C112	186	1	83	1	5	1	90			1	62	1	32	1	83	1	71	0
C6	187	1	83	1	0	1	58			1	83	1	70	1	90	1	92	0
C44	187	1	83	1	7	1	92	1	52	1	74	1	58	1	86			0
C44	188	1	88	1	6	1	89			1	83	1	87	1	87	1	90	0
C1	188	1	88	1	6	1	61			1	60	1	51	1	61	1	60	0
C45	188	1	97	1	2	1	98			1	71	1	40					0
C46	188	1	97	1	5	1	91	1	86	1	81	1	63	1	91	1	91	0
C41	189	1	98	1	2	1	94	1	86	1	86	1	67	1	76	1	92	0
C130	189	1	88	1	2	1	91			1	81	1	36	1	89	1	86	0
C90	190	1	83	1	0	1	88	1	82	1	89	1	85	1	87	1	82	0
C7	190	1	87	1	3	1	90			1	83	1	69	1	87	1	85	0
C3	190	1	83	1	2	1	92	1	87	1	92	1	91	1	92	1	92	0
C100	190	1	83	1	0	1	79	1	74	1	72	1	71	1	80	1	78	0
C6	191	1	88	1	2	1	90			1	55	1	31	1	82	2	54	0
C16	192	1	95	1	4	1	100			1	96	1	98	1	98	1	98	0
C102	192	1	90	1	1	1	96	1	88	1	95	1	96	1	96	2	14	0
C101	193	1	90	1	2	1	92	1	91	1	88	1	67	1	89	1	93	0
C103	193	1	73	1	0	1	86		59	1	76	1	39	1	87	2	22	0
C103	193															2	33	
C11	193	1	88	1	1	1	94			1	95	1	72	1	96	1	94	0
C1	193	1	85	1	4	1	91	1	65	1	89	1	81	1	89	1	80	0
C11	193	1	88	1	1	1	94			1	95	1	72			1	94	0
C2	195	1	91	1	1	1	92			1	88	1	85	1	87	1	93	0
C81	196	1	83	1	1	1	88			1	87	1	70	1	89	1	89	0
C131	196	1	93	1	3	1	100			1	95	1	60	1	96	1	98	0
C8	197	1	87	1	2	1	94	1	88	1	86	1	87	1	92	1	90	0
C3	197	1	81	1	1	1	89			1	86	1	89	1	90	1	88	0
C7	197	1	85	1	1	1	93	1	82	1	91	1	65	1	91	1	92	0
C132	198	1	86	1	1	1	93	1	95	1	91	1	93	1	94	1	93	0
C3	198	1	81	1	1	1	89			1	86	1	89	1	90	1	88	0
C3	198	1	88	1	3	1	92					1	92	1	93	1	91	0
C55	198	1	99	1	1	1	94			1	86	1	52					0
C1	199	1	85	1	2	1	88	1	86	1	90	1	86	1	89	1	88	
C3	199	1	84	1	2	1	87			1	88	1	88	1	84	1	86	0
C83	199	1	74	1	3	1	90	1	75	1	50	1	28	1	88	1	86	0

C82	200	1	77	1	2	1	83			1	54			1	81	2	20	0 (low U)
C82	200															2	23	
C82	200															2	20	
C84	200	1	96	1	0	1	99			1	97	1	87	1	99			0
C51	200	1	98	1	3	1	92	1	93	1	79	1	59	1	89	1	93	0
C46	200	1	95	1	3	1	97	1	72	1	56	1	39	1	95	1	89	0
C46	200	1	100	1	0	1	97			1	71	1	62	1	85	1	85	0
C85	201	1	100	1	3	1	97	1	71	1	73	1	53	1	94	`	94	0
C85	201								78									
C1	201	1	75	1	1	1	88	1	58	1	78	1	27	1	83	1	86	0
C133	202	1	89	1	3	1	92			1	86	1	73	1	94	1	93	0
C134	202	1	91	1	2	1	93			1	52	1	15			2	48	0 (low U)
C50	202	1	91	1	2	1	93			1	52	1	15	2	6	2	20	0
C68	203	1	96	1	1	1	92					1	90					0
C68	203	1	92	1	1	1	93	1	85	1	80	1	73	1	90	1	87	0
C69	203	1	86	1	0	1	97			1	95	1	50	1	96	1	90	0
C5	203	1	80	1	1	1	86			1	80	1	70	1	83	1	84	0
C51	204	1	99	1	2	1	94	1	93	1	90	1	49	1	94	1	91	0
C116	204	1	95	1	3	1	97			1	91	1	59	1	95	1	90	0
C6	205	1	88	1	0	1	92	1	91	1	91	1	89	1	94	1	90	0
C2	206	1	95	1	2	1	97			1	73	1	44					0
C104	206	1	90	1	4	1	89	1	88	1	84	1	70	1	85	1	89	0
C2	206	1	77	1	2	1	83					1	54	1	81	2	20	0
C18	208	1	87	1	1	1	91	1	93	1	93	1	92	1	52	1	92	0
C2	208	1	89	1	4	1	97	1	92	1	88	1	54	1	97	1	85	0
C3	208	1	87	1	3	1	90	1	88	1	88	1	89	1	89	1	89	0
C24	208	1	83	1	1	1	89	1	89	1	79	1	70	1	90	1	89	0
C3	208	1	87	1	3	1	90	1	88	1	88	1	89	1	89	1	89	0
C106	208	1	99	1	2	1	93			1	89	1	83	1	92	1	90	0
C7	209	1	85	1	5	1	91			1	87	1	86	1	87	1	86	0
C6	210	1	97	1	4	1	96	1	71	1	89	1	74	1	95	1	90	0
C107	210	1	97	1	4	1	94			1	68	1	32	1	91	1	90	0
C108	210	1	97	1	0	1	92					1	83					0
C3	210	1	83	1	8	1	91	1	91	1	90	1	89	1	88	1	89	0
C6	210	1	81	1	2	1	94	1	93	1	93	1	92	1	93	1	93	0
C111	210	1	83	1	2	1	94	1	93	1	91	1	94	1	93	1	92	0
C77	211	1	82	1	1	1	90	1	77	1	81	1	53	2	32	1	88	0
C3	212	1	84	1	4	1	92		91	1	89	1	91	1	91	1	91	0
C109	213	1	77	1	8	1	94			1	83	1	74	1	85	1	85	0
C109	213			1	3													
C109	213			1	6													
C11	213	1	80	1	1	1	90			1	89	1	88	1	86	1	86	0
C6	215	1	88	1	3	1	90			1	63	1	36	1	88	1	88	0
C2	215	1	88	1	0	1	91			1	83	1	46	1	93	1	90	0
C1	216	1	92	1	2	1	89	1	93	1	94	1	93	1	94	1	94	0
C110	217	1	82	1	1	1	88	1	86	1	84	1	78	1	87	1	88	0
C7	217	1	78	1	0	1	94			1	91	1	46	1	94	1	92	0
C85	218	1	93	1	2	1	94			1	90	1	30	1	90	1	91	0
C87	218	1	72			1	77			1	56	1	24			1	71	0
C4	218	1	86	1	3	1	82	1	86	1	87	1	67	1	89	1	78	0
C86	218	1	72			1	77			1	56	1	24			1	71	0
C7	219	1	84	1	2	1	84			1	64	1	38	1	90	1	88	0
C7	219													1	90			
C7	219													1	89			
C7	219													1	90			
C88	219	1	88	1	1	1	91	1	93	1	92	1	90	1	91	1	89	0

C34	219	1	89	1	1	1	92	1	71	1	89	1	34	1	93	11	93	0
C89	219	1	86	1	3	1	91			1	50	1	29	1	89	1	88	0
C25	220	1	95	1	4	1	94			1	89	1	41	1	92	1	92	0
C17	220	1	89	1	4	1	91	1	98	1	92	1	91	1	93	1	91	0
C82	220	1	90	1	1	1	98	1	97	1	97	1	95	1	98	1	96	0
C3	220	1	84	1	3	1	82			1	77	1	58	1	90	1	89	0
C18	222	1	77	1	4	1	98	1	97	1	96	1	96	1	98	1	95	0
C12	223	1	85	1	1	1	91	1	94	1	88	1	88	1	92	1	89	0
C90	223	1	87	1	2	1	90	1	92	1	94	1	96	1	95	1	93	0
C1	223	1	100	1	4	1	96			1	90	1	94	1	84	1	94	0
C31	224	1	94	1	2	1	99					1	31					0
C1	224	1	86	1	2	1	93			1	89	1	86	1	91	1	89	0
C69	224	1	78	1	0	1	92	1	79	1	86	1	82	1	93	1	91	0
C3	224	1	85	1	2	1	87			1	91	1	91	1	91	1	88	0
C91	226	1	85	1	0	1	89			1	90	1	90	1	89	1	88	0
C4	226	1	94	1	4	1	95			1	91	1	86	1	90	1	94	0
C5	227	1	97	1	7	1	99			1	76	1	38	1	98	1	93	0
C2	227	1	93	1	6	1	94	1	92	1	92	1	87	1	95	1	84	0
C12	228	1	79	1	1	1	91	1	90	1	89	1	88	1	93	1	90	0
C4	230	1	94	1	3	1	91	1	82	1	87	1	65	1	89	1	90	0
C43	230	1	70	1	3	1	80			1	47	1	34	1	76	1	75	0
C92	230	1	92	1	4	1	93			1	86	1	84	1	93			0
C2	230	1	82	1	2	1	92			1	85	1	82	1	91	1	80	0
C16	230	1	90	1	0	1	90	1	91	1	90	1	85	1	92	1	90	0
C5	232	1	89	2	1	1	96	1	91	1	87	1	74	1	96	1	90	0
C9	233	1	76	1	1	1	89	1	67	1	48	1	32	1	92	2	7	0
C8	233	1	90	1	3	1	98	1	95	1	95	1	96	1	96	1	95	0
C65	233	1	80	1	1	1	90			1	91	1	91	1	90	1	90	0
C93	233	1	88	1	3	1	92	1	87	1	80	1	63	1	89	1	91	0
C44	233					1	86											
C61	234	1	85	1	1	1	90			1	90	1	85	1	91	1	92	0
C62	235	1	91	1	2	1	93	1	91	1	86	1	31	1	92	1	91	0
C135	235	1	91	1	1	1	94			1	89	1	81	1	95	1	91	0
C3	235	1	83	1	4	1	92	1	90	1	90	1	92	1	89	1	91	0
C3	237	1	86	1	2	1	91	1	91	1	92	1	89	1	93	1	90	0
C63	237	1	85	1	2	1	95	1	93	1	94	1	94	1	94	1	93	0
C32	237	1	79	1	2	1	86	1	91	1	90	1	91	1	89	1	88	0
C64	238	1	80	1	2	1	92			1	90	1	76	1	91	1	90	0
C8	238	1	91	1	3	1	95			1	93	1	95	1	94	1	94	0
C10	239	1	98	1	4	1	95	1	90	1	86	1	90	1	94	1	91	0
C1	239	1	95	1	2	1	93			1	95	1	56					0
C1	239 `											1	67					

Control samples with 81-140 X 10^9 platelets/L

Control code	adj PRP Plt $x10^9$/L	Information on whether agonist responses were normal (0) or abnormal (1) for the sample platelet count and the % maximal aggregation (MA) — 1.25 mg/mL ristocetin Result	1.25 mg/mL ristocetin MA	0.5 mg/mL ristocetin Result	0.5 mg/mL ristocetin MA	5 µg/ml Collagen Result	5 µg/ml Collagen MA	5 µM ADP Result	5 µM ADP MA	LTA findings 0=normal/non-diagnostic 1=abnormal (specify)
C21	80	1	72	1	4	1	72	1	62	0
C37	83	1	74	1	4	2	37	2	10	1
C37	83					2	29	2	11	(ADP and Col)
C39	83	1	81	1	6	1	87	1	63	0
C37	83	1	74	1	4	2	37			0
C22	83	1	80	1	10	1	67			0
C52	83	1	95	1	4	1	84			0
C123	85	1	85	1	4	1	92	1	46	0
C17	85	1	91	1	3	1	92			0
C53	85	1	79	1	6	1	88	1	52	0
C3	86	1	83	1	2	1	92	1	66	0
C7	87	2	58	1	3	1	83	1	65	1 (ristocetin false positive)
C47	88	1	97	1	5	1	91			0
C124	90	1	84	1	0	1	89	1	80	0
C6	90	1	86	1	4	1	76			0
C48	90	1	92	1	4	1	91	1	48	0
C48	90	1	95	1	6					
C48	90	1	96	1	4	1	91			0
C8	91	1	78	1	1	1	85	1	49	0
C11	91	1	88	1	5	1	80	1	82	0
C19	91	1	88	1	5	1	80	1	82	0
C6	92	1	64	1	1	1	76	1	52	0

C1	92	1	74	1	3	1	72	1	44	0
C1	92						68			
C1	92					2 outlier	59			
C125	93	1	94	1	3	1	63	2	34	0
C54	96	1	86	1	5	1	81			0
C115	98	1	99	1	6	1	74			0
C118	99	1	85	1	5	1	87	1	87	0
C113	100	1	91	1	3	1	88	1	86	0
C4	100	1	97	1	2	1	91			0
C23	100	1	95	1	2	1	96			0
C1	100	1	85	1	1	1	71	1	32	0
C115	100	1	83	1	2	1	87	1	31	0
C2	100	1	88	1	1	1	93	1	77	0
C3	100	1	118	1	0	1	112	1	94	0
C6	102	1	60	1	1	1	88	1	53	0
C56	102	1	103	1	4	1	100			0
C5	103	1	106	1	4	1	103			0
C57	103	1	86	1	2	1	94			0
C1	104	1	75	1	4	1	73	1	58	0
C51	105	1	85	1	2	1	87			0
C8	105	1	93	1	1	1	87	1	68	0
C11	105	1	91	1	8	1	103	1	66	0
C7	106	1	79	1	2	1	89	1	45	0
C1	106	1	82	1	12	1	83	1	63	0
C1	107	1	72	1	1	1	79	1	44	0
C74	108	1	91	1	3	1	90	1	44	0
C67	108	1	85	1	0	1	87	1	84	0
C51	108	1	93	1	10					0
C6	109							1	93	0
C6	109							1	77	
C48	109	1	143	1	3	1	151	1	95	0
C16	109	1	78	1	1	1	86	1	84	0

C49	109	1	86	1	2	1	64	1	59	0
C50	110	1	90	1	3	1	123			0
C67	110	1	79	1	5	1	87	1	57	0
C82	111	1	86	1	9	1	82	1	71	0
C85	111	1	84	1	4	1	73	1	43	0
C1	112	1	80	1	5	1	86	1	71	0
C105	112	1	99	1	4	1	92	1	90	0
C105	112					1	91			
C11	115	1	85	1	1	1	83	1	71	0
C16	116	1	87	1	8	1	92	1	91	0
C23	116	1	92	1	3	1	89			0
C15	116	1	96	1	5	1	89			0
C10	116	1	91	1	3	1	89			0
C7	117	1	90	1	0	1	87	1	85	0
C90	117	1	89	1	1	1	87	1	72	0
C126	118	2	53	1	1	1	81	1	46	0
C2	118	1	86	1	0	1	85	1	65	0
C2	118	1	96	1	5	1	90			0
C52	119	1	100	1	1	1	100	1	61	0
C25	119	1	88			1	89			0
C24	119	1	79	1	11	1	77	1	53	0
C24	119	1	81	1	3	1	76	1	58	
C70	120	1	82	1	2	1	87			0
C127	120	1	88	1	0	1	91	1	98	0
C49	120	1	89	1	2	1	90	1	77	0
C1	121	1	81	1	1	1	87	1	87	0
C1	121	1	88	1	2	1	94	1	93	
C71	121	1	96	1	2	1	89			0
C5	122	1	80	1	6	1	73	1	44	0
C72	123	1	87	1	3	1	86			0
C2	123	1	88	1	1	1	96	1	82	0
C2	124	1	87	1	1	1	92	1	70	0

C1	125	1	75	1	1	1	86	1	84	0
C73	125	1	83	1	2	1	86			0
C15	125	1	84	1	4	1	85			0
C8	126	1	82	1	0	1	91	1	70	0
C47	126	1	77	1	5	1	84	1	74	0
C115	126	1	82	1	0	1	91	1	70	0
C74	127	1	90	1	2	1	94	1	59	0
C1	128	1	83	1	6	1	87	1	65	0
C75	128	1	74	1	2	1	86	1	43	0
C2	129	1	84	1	0	1	84	1	75	0
C7	129	1	80	1	2	1	89	1	83	0
C6	130	1	82	1	0	1	88	1	82	0
C23	130	1	96	1	6	1	100			0
C76	130	1	91	1	2	1	90			0
C22	130	1	93	1	10	1	86			0
C1	131	1	91	1	7	1	99			0
C42	131	1	95	1	2	1	86	1	83	0
C11	131	1	78	1	1	1	85	1	42	0
C2	132	1	79	1	1	1	85	1	71	0
C77	132	1	88	1	0	1	92	1	86	0
C17	134	1	94	1	4	1	96			0
C114	135	1	87	1	2	1	91			0
C10	135	1	97	1	4	1	94			0
C49	137	1	80	1	1	1	92	1	58	0
C1	138	1	94	1	4	1	94			0
C1	138	1	90	1	4	1	91			0
C1	139	1	85	1	3	1	88			0
C22	139	1	83	1	6	1	82			0
C128	139	1	82	1	1	1	86	1	86	0
C49	139	1	95	1	4	1	88	1	76	0

Control samples with ≤80 X 10^9 platelets/L

Control code	adj PRP Plt x10^9/L	1.25 Risto 0=NA 1=Normal 2=Reduced	1.25 Risto MA	0.5 Risto 0=NA 1=Normal 2=Increase	0.5 Risto MA	5 Col 0=NA 1=Normal 2=Reduced	5 Col MA	LTA findings 0=Normal/non-diagnostic 1=Abnormal (specify)
C17	10	1	64	1	11	1	28	0
C19	17	1	72	1	12	1	58	0
C16	19	1	88	1	0	1	94	0
C1	19	1	88	1	0	1	89	0
C2	19	2	44	1	3	2	32	0
C122	20	1	64	1	4			0
C121	23	1	72	1	2	1	87	0
C12	28	2	46	1	1	1	62	0
C34	31	1	64	1	12			0
C34	31	2	50	1	7			0
C120	34	1	72	1	8	1	82	0
C23	35	1	100	1	11	1	96	0
C3	36	1	46	1	8	1	49	0
C3	36			1	8			
C3	36			1	3			
C11	38	1	58	1	6	1	62	0
C13	40	2	11	1	6	1	56	0
C140	42	1	58	1	8	1	53	0
C4	42	2	39	1	0	2	44	0
C117	43	1	67	1	6			0
C117	43	1	69					
C3	44	1	75	1	4	1	66	0

C20	45	1	70	1	2	1	70	0
C1	46	1	76	1	9	1	60	0
C6	47	1	70	1	1	1	54	0
C20	47	1	78	1	4	1	91	0
C115	50	1	66	1	2	1	69	0
C1	50	1	62	1	16	1	61	0
C3	50	2	52	2	20	2	44	1 (failed all agonists)
C119	50	1	58					0
C119	50	1	51					
C23	50	1	112	1	6	1	118	0
C8	50	1	106	1	3	1	98	0
C25	53	1	94	2	20	2	47	0
C6	53	1	49	1	0	1	59	0
C6	53					1	55	
C139	53	1	73	1	2	2	33	0
C1	55	1	69	1	9	1	61	0
C6	55	1	59	1	7	1	66	0
C7	56	1	62	1	1	1	68	0
C27	56	2	42	1	1	2	33	1 (collagen and ristocetin)
C27	56	2	30	1	2	2	29	
C1	58	1	71	2	21	1	67	0
C1	58	1	80	1	14	1	71	
C11	58	1	71	1	1	1	76	0
C2	59	2	54	1	1	1	78	0
C11	60	1	71	1	9	1	67	0
C12	61	1	86	1	1	1	97	0
C28	62	1	79	1	3	1	72	0
C29	64	1	98	1	6	1	95	0
C137	65	1	54	1	4			0
C8	65	1	86	1	2	1	87	0
C4	69	1	72	1	2	1	80	0

C21	70	1	79	1	3	1	85	0
C30	70	1	68	1	1	1	77	0
C1	72	1	74	1	7	1	74	0
C1	72			1	3			
C2	73	1	78	1	0	1	86	0
C1	76	1	87	1	6	1	74	0
C33	78	1	91	1	6	1	90	0
C1	79	1	64	1	3			0
C8	80	1	89	1	2	1	96	0
C35	80	1	75	1	4	1	86	0
